# Mechanical Activation by Ball Milling as a Strategy to Prepare Highly Soluble Pharmaceutical Formulations in the Form of Co-Amorphous, Co-Crystals, or Polymorphs

**DOI:** 10.3390/pharmaceutics14102003

**Published:** 2022-09-21

**Authors:** Luz María Martínez, Jorge Cruz-Angeles, Mónica Vázquez-Dávila, Eduardo Martínez, Paulina Cabada, Columba Navarrete-Bernal, Flor Cortez

**Affiliations:** Tecnologico de Monterrey, School of Engineering and Sciences, Ave. Eugenio Garza Sada 2501 Sur, Monterrey 64849, NL, Mexico

**Keywords:** drug, amorphous, milling, co-crystals, polymorphs, mechanical activation

## Abstract

Almost half of orally administered active pharmaceutical ingredients (APIs) have low solubility, which affects their bioavailability. In the last two decades, several alternatives have been proposed to modify the crystalline structure of APIs to improve their solubility; these strategies consist of inducing supramolecular structural changes in the active pharmaceutical ingredients, such as the amorphization and preparation of co-crystals or polymorphs. Since many APIs are thermosensitive, non-thermal emerging alternative techniques, such as mechanical activation by milling, have become increasingly common as a preparation method for drug formulations. This review summarizes the recent research in preparing pharmaceutical formulations (co-amorphous, co-crystals, and polymorphs) through ball milling to enhance the physicochemical properties of active pharmaceutical ingredients. This report includes detailed experimental milling conditions (instrumentation, temperature, time, solvent, etc.), as well as solubility, bioavailability, structural, and thermal stability data. The results and description of characterization techniques to determine the structural modifications resulting from transforming a pure crystalline API into a co-crystal, polymorph, or co-amorphous system are presented. Additionally, the characterization methodologies and results of intermolecular interactions induced by mechanical activation are discussed to explain the properties of the pharmaceutical formulations obtained after the ball milling process.

## 1. Introduction

Almost half of the oral administered commercial drugs have low solubility, which affects their bioavailability [1,2]. Several alternatives to modify the supramolecular structure of APIs have been proposed to overcome their low solubility; these strategies include amorphization [3,4,5], solid dispersion [6,7,8,9], preparation of co-crystals [10,11], and polymorphs [12,13,14], among others. These approaches to enhance solubility involve non-covalent interactions, such as the electrostatic or intermolecular interactions between API molecules and the components of pharmaceutical formulations. Non-covalent interactions are preferred because they do not alter the pharmacological activity of the APIs. The selection of each strategy to improve the drugs’ properties depends on the particular API’s chemical nature. Preparation methodologies of drug formulations also depend on API properties, such as structural and thermal stability. Considering that many APIs are thermosensitive, non-thermal emerging alternative techniques, such as mechanical activation or milling, have become an increasingly common preparation method for co-amorphous, co-crystals, and polymorph drugs.

Several publications present overviews of specific applications of milling for the development of pharmaceutical products. In 2013, Braga et al. [15] presented a summary of scientific literature on the preparation of only co-crystals, while Einfal et al. [16] published, in the same year, a summary of amorphization of APIs by milling. Furthermore, in 2015 an overview of different milling techniques for improving the solubility of poorly water-soluble drugs was published [17]; this last article covered different types of milling, but focused its analysis on particle size reduction. Although these reviews are complete within their specific scopes, the authors of the present work believe that ball milling is a technique that has become one of the most widely used methods to enhance a drug’s physicochemical properties. For this reason, a summary of recent research in preparing and characterizing pharmaceutical formulations through ball milling to improve APIs’ physical-chemical properties is worth an update on this topic.

The present review summarizes the most representative studies that applied ball milling to obtain different formulations with the enhanced properties of either co-crystal or co-amorphous systems, using low molecular weight components and polymorphs. First, a general description of these types of formulations is presented. Then, an analysis and comparison of the available information of milling conditions reported and their effects on improving drug properties are discussed. Unlike previously published reviews, this is the only work in which the solubility, phase transitions, structural stability, and characterization results of intermolecular interactions induced by mechanical activation are compared and presented together for co-crystals, co-amorphs, and polymorphs drugs.

## 2. Pharmaceutical Formulations Based on Structural Properties

### 2.1. Amorphous Pharmaceutical Formulations Prepared by Milling

An amorphous solid has no long-range order of molecular packing and lacks a well-defined molecular conformation. Amorphization has been introduced as a promising alternative to enhance drugs’ solubility in the last two decades. It has been demonstrated that amorphous materials usually have a higher solubility and dissolution rate than their crystalline state [18,19]. The enhancement of solubility in amorphous materials can be explained, in terms of the ease of overcoming intermolecular forces [20,21,22]. One of the most common techniques to achieve amorphization is the process of melt quenching. This process consists of melting a crystalline sample and then proceeding to rapid cooling, thus obtaining the amorphous state [23,24,25]. This method presents disadvantages for thermosensitive drugs, since the high temperatures required to achieve melting may result in thermal decomposition. The study performed by Wlodarski et al. [26] is a clear example of the wide range of thermosensitive drugs that currently exist with low solubility that cannot be obtained in the amorphous state by melt quenching. Due to this drawback, mechanical stress is a non-thermal alternative introduced for amorphization. It has been proven that milling allows for the transformations of the solid crystalline state of matter, thus causing a shift from the crystalline form to the amorphous state [27,28]. The milling process consists of decreasing the compound particle size, thus promoting the accumulation of energy to such a degree that it goes over the critical value that causes a structural deformation of the crystalline structure, which results in the amorphization of the material [29]. However, due to having higher entropy and free energy than the corresponding crystals, the amorphous state is inherently unstable, and recrystallization may occur [30]. The preparation of binary systems forming intermolecular interactions has been reported to avoid recrystallization [30,31,32,33]. The selection of a co-former to obtain a co-amorphous system can be a second drug or an excipient, such as sugars, organic acids, amino acids, or surfactants [34,35,36,37]. For the reviewed studies in this work, the milling process for amorphization is solely reported under drying conditions. It has been observed that the addition of a solvent in the milling process tends to induce co-crystallization [38].

Besides amorphization, it is important to understand that ball milling is a technique that can lead to the formation of a microcrystalline (or nanocrystalline) state, where this last state involves particle size reduction without the deformation of the crystalline structure. Microcrystallinity results in an increased surface area, higher drug solubility, and increased dissolution rate [39].

There are multiple techniques, such as X-ray diffraction, dynamic light scattering, infrared and Raman spectroscopy, differential scanning calorimetry, and scanning electron microscopy, that are useful techniques for differentiating the microcrystalline and analysis of amorphous states. The following section presents drug formulations in the form of co-crystals.

### 2.2. Drug Co-Crystals Prepared by Mechanical Activation

Another strategy to enhance solubility with the mixtures of two components is the formation of co-crystals. Co-crystals have acquired different definitions over the years; generally, a co-crystal is a solid material composed of two or more molecules in the same crystal lattice.

**Figure 1:**
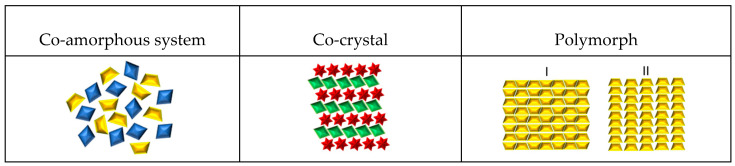
Schematic representation of API formulations: co-amorphous system, co-crystal, and polymorph.

Pharmaceutical co-crystals are crystalline single-phase materials composed of two or more compounds. Co-crystals typically consist of an API and one or more additional molecular or ionic compounds called “co-formers” that are kept together via hydrogen bond or electrostatic interactions [10,40,41,42]. A cocrystal has a different crystal structure to either of the starting materials and, as a result, different physicochemical properties [43]. Figure 1 shows a schematic representation of a co-crystal structure, compared with a co-amorphous system and polymorph. Co-crystals are prepared by different methods, such as the supercritical anti-solvent (SAS) process [44], extrusion [45], freeze-drying [46], spray drying [47], and laser radiation [48]. However, chemical integrity is not always maintained with these preparation methodologies. Some limitations are sometimes encountered, like solubility of the components in a given solvent or solvent mixture and thermal degradation. As a counterpart, mechanochemical methods have also proven effective for co-crystal formation; the preparation of co-crystal by mechanical activation can be achieved by dry and liquid-assisted grinding [49,50,51]. Several studies report the preparation of co-crystals by grinding with a mortar [52,53]. However, those results are not included in this review.

### 2.3. Drug Polymorphs as a Result of the Milling Process

It is estimated that about 80–90% of organic compounds are polymorphic [54]. Polymorphic solids exist in multiple crystalline solid forms [55,56,57,58]. It is well-known that changing the arrangement of atoms, molecules, or ions within a crystalline lattice raises the differences in physicochemical properties, including the solubility and bioavailability [59]. Therapeutic efficacy is also affected by structural arrangements [54]. One example of a polymorphism affecting drug properties is when a drug interconverts into more and less soluble forms, thus limiting its absorption and bioavailability [12]. There is a wide range of methodologies to prepare polymorphs: crystallization from a single or mixed solvent [60], exposure to organic vapor [61], dehydration of solvates by heat or by slurry [62], seeding [63], laser-induced [64], or supercritical fluid crystallization [65] are some of these preparation methods. However, this review is focused on the obtention of polymorphic forms using ball milling. The occurrence of polymorphism is not limited to single component formulations, but its existence has also been documented in multicomponent systems, such as co-crystals, salts, solvates, and hydrates [57]. Some examples are addressed later in this review.

Below are some of the schematic representations of the previously described systems (see Figure 1).

Various factors can individually change and influence the final characteristics of an active pharmaceutical ingredient after milling. Therefore, it is necessary to identify the prevailing conditions under which amorphous systems, co-crystals, and polymorphs are obtained using griding or milling. In the following sections, the analyses of each experimental condition are presented.

## 3. Factors Affecting Drug Formulations during the Mechanical Activation Process

Table 1, Table 2 and Table 3 present an overview of the experimental milling conditions, such as the instrument (type of mill), solvent, time, and temperature, which are reported for each type of drug formulation. The first column contains a code with one number and a letter identifying each drug formulation in all tables. In each code, the number refers to a consecutive numeration of the article reviewed, and the letter stands for the following criteria: A, amorphous; C, co-crystal; and P, polymorph.

### 3.1. Ball Milling Instruments

After reviewing the information presented in Table 1, Table 2 and Table 3, it can be inferred that a planetary ball mill is the type of mill most commonly used in all three types of drug formulations. Planetary instruments have vessels placed inside a rotating disk and can induce high energy to the powder to prompt changes. Zirconium oxide (ZrO_2_) and stainless-steel milling jars are the most common cells used for polymorphs and amorphous, whereas stainless steel alone is the most used for co-crystals. In most cases, the milling jar material is the same as the milling balls, except for the work of co-crystals reported by Stolar et al. [66], who use a different material: polymethylmethacrylate for the milling jar and stainless steel for the balls. Only Manin et al. [67] report the use of agate. For oscillatory/vibrational mills, the milling speed ranges from 10 to 30 Hz for all drug formulations. The most common speed for amorphous and co-crystals is 30 Hz. No trend is observed for polymorphs. In planetary mills, values reported ranges from 4.2 to 10.8 Hz for amorphous, with 6.7 Hz being the most common value for all formulations (amorphous, polymorphs, and co-crystals).

### 3.2. Temperature during the Milling Process

From Table 1, it was observed that, for amorphous systems, most milling processes were carried out in cold conditions (4–6 °C) or cryogenic temperatures (cell dips in liquid nitrogen), whereas for co-crystals, the temperature commonly used for grinding was room temperature. For polymorphs, the milling temperatures reported range from cryogenic temperature to 130 °C, although room temperature was the most common condition (see Table 2 and Table 3).

### 3.3. Phase Transformation Mechanism by Ball Milling and Temperature Effect

The process of amorphization by milling can be explained from different perspectives. One of them indicates that, when a crystalline material is milled under direct collision, the first thing that is caused is the reduction of the material’s particle size, which is accompanied by changes in morphology and crystallinity. Understanding that if this milling process is carried out below the glass transition temperature (Tg) of the material (because, at this point, the molecular mobility decreases), amorphization is facilitated [16,17,27,68,69].

For co-crystallization there are three accepted mechanisms using grinding methods, i.e., molecular diffusion, and eutectic formation, which are mediated by an amorphous phase. The molecular diffusion mechanism is representative of the solvent/liquid-assisted grinding method. When drops of solvent are used for a mixture with components that are similar, in terms of solubility, the liquid solvent serves as a medium for promoting molecular diffusion and facilitating the interaction between the drug and co-former [15]. Moreover, the eutectic co-crystallization mechanism suggests that, when two solids are in physical contact by grinding at the eutectic temperature, there is a liquid phase formation, where the solid remains from both original crystals work as seeds for the co-crystallization process. [70,71,72]. Lastly, grinding can also induce enough disorder in solid mixtures to promote an amorphous phase formation. Storage or milling conditions, such as solvents and water presence, can increase molecular mobility and promote the co-crystallization of previously formed amorphous phases [73].

The polymorph formation mechanism upon milling is strongly related to several factors induced by the mechanical stress of high-energy milling. The main factors are temperature and microstructural changes, such as the size of crystallites, crystalline defects, and lattice distortions; these factors are believed to work collectively.

As previously mentioned in the mechanism for amorphization by milling, when milling occurs below the glass transition temperature, the material leads to amorphization; however, when milling occurs at a temperature above Tg, the material leads to polymorphic transformations, whereby in the formation of polymorphs by grinding the amorphous state is an intermediate state [74,75].

In addition to temperature, experimental work shows that a certain extent of defects in the system are necessary to trigger the polymorphic transformation. For most crystalline compounds, the stress applied during mechanical milling can create new defects in their crystal lattices and contribute to lattice disorder. The nucleation and growth of the new lattice defects formed within the structure may result in solid-state polymorphic interconversion upon milling [75,76]. Evidence of these factors affecting the formation of polymorphs is the study of the conversion of ranitidine hydrochloride from form 1 into form 2 [74]. Grinding of form 1 generates large amounts of heat and vibrational energy, giving rise to grinding-induced crystal lattice disruption or process-induced disorder. The formation of an amorphous intermediate follows the elimination of form 1 crystals. Finally, through continuous milling, form 2 nuclei are produced.

An analysis of experimental data related to the temperature effect during phase transformation by milling is shown in Table 1. It was observed that, for amorphous systems, most milling processes were carried out in cold conditions (4–6 °C) or cryogenic temperatures (cell dips in liquid nitrogen). This is consistent with the mechanism proposed, in which it was established that amorphization occurs at a temperature below the glass transition temperature. For co-crystals, the temperature commonly used for grinding was room temperature. This could be explained because mechanical activation generates heat during milling, and the sample is exposed to temperatures near or above the glass transition temperature. For polymorphs, the milling temperatures reported ranges from cryogenic temperature to 130 °C, although room temperature was the most common condition (see Table 2 and Table 3).

### 3.4. Solvent Effect

Dry ball milling (DBM) is when a sample is subjected to the milling procedure under dry conditions. Terms such as “wet grinding”, “solvent-drop grinding”, “liquid assisted grinding”, and “kneading” all imply that a solvent is involved, whether by intention or not (air humidity) [15]. In 2006, Friscić et al. changed the solvent drop grinding term into liquid-assisted grinding (LAG) [77], which became the most frequently used expression to indicate a grinding process with a tiny amount of solvent [15]. According to Table 1, Table 2 and Table 3, most studies prepared the formulation by adding a solvent to induce co-crystallization. In contrast, co-amorphous and polymorphs were mainly obtained under dry conditions. Additionally, it has been observed that the addition of a small amount of solvent increases the rate of co-crystallization [51] by a process called solution-mediated phase transformation [78]. Therefore, most co-crystals require adding a particular solvent to improve the miscibility of the drug and co-formers. Whereas, for polymorphs, adding a solvent also allows for accessibility to new metastable forms and a shorter experimental time to obtain new polymorphs [79]. It has been shown that the chemical properties of the solvent can lead to a specific polymorph [79,80,81,82,83].

### 3.5. Effect Changing Composition

Most of the co-crystals prepared by milling use the 1:1 molar ratio; from all the articles reviewed, just five studies prepared co-crystals using molar ratios of 2:1 or 1:2. A similar situation was observed for co-amorphous formulations, although it was common to find studies with molar ratios 1:1, 1:2, and 2:1. Just one study reported a formulation with a molar ratio 1:4 and 1:5 (see Table 1).

### 3.6. Milling Time

Table 1, Table 2 and Table 3 show that adequate milling time to produce an intended structural change varies between studies. When a thermosensitive drug is subjected to milling, it is necessary to program pauses at specific times to maintain low temperatures. Nonetheless, there are studies with no thermosensitive drugs that have reported milling times between 30 to 180 min with no breaks.

For the preparation of co-crystals, short periods between 20 to 60 min are reported, although one study reported 5 h [44]. Milling time for polymorphs is longer than for co-crystals; usually, the required time is longer than one hour, and one study even lasted 10 h [34]. Moreover, when there are more than two polymorphic structures of the compound, the increase in milling time can lead to several transformations or what is called two-step polymorphisms.

For co-amorphous, the milling time varies, depending on the type of mill and milling temperature; however, the most common time range is between 60 and 180 min.

In all drug formulations studied here, a difficulty emerges in characterizing all of the properties of the drug formulations obtained by milling with one single analytical method. As a result, in an effort to study their enhanced properties, a wide number of characterization techniques are used to study them. The most used techniques for characterization in all drug formulations (amorphs, co-crystals, and polymorphs) are XRD and thermal techniques, followed by FT-IR. That is the main reason why this review focuses on a detailed analysis of characterization results and the primary information that can be obtained from each characterization method.

**Table 1:** Conditions of preparation of co-amorphs by ball milling method.

#	Drug 1	Drug 2 Molar-Ratio	Amorphous Stability (Storage-Conditions)	Mill Type	Volume Cell Material	Balls-Num. Material and Sample Weight	Milling Frequency	Milling Temp. (°C)	Milling Time	Ref.
1A	Mebendazole	Twenty different amino acids 1:1	Not reported	Oscillatory ball mill	25 mL Jar	2 (d = 12 mm) stainless steel balls 1000 mg	30 Hz	Not specified	1, 5, 15, 30, and 60 min	[84]
	Carvedilol									
	Carbamazepine									
	Simvastatin									
	Indomethacin									
	Furosemide									
2A	Furosemide	Arginine	Dry conditions at 25 °C or 40 °C for 15 months of storage	Oscillatory ball mill	25 mL Jar	2 (d = 12 mm) stainless steel balls 750 mg	30 Hz	5 °C	180 min	[85]
	Nitrofurantoin									
	Cimetidine	Citrulline								
	Mebendazole									
3A	Sulfathiazole	Polyvinylpyrrolidone Xpvp: 0.6 and 0.7	Storage at 4 °C over a year	Planetary mill	50 cm^3^ ZrO_2_ milling jars	3 balls (d = 20 mm) ZrO_2_. 2.5 g	6.6 Hz	Room temperature	10 h (15 h total) 10 min pauses after every 20 min	[86]
	Sulfadimidine									
4A	Naproxen	Cimetidine 1:2, 1:1, 2:1	Dry conditions at 4, 25 and 40 °C for up to 33 days or further extended to 186 days	Oscillatory ball mill	25 mL stainless steel milling jar	2 (d = 12 mm) stainless steel balls 1 g of sample per grinding cell	30 Hz	4 °C ± 2 °C	60 min	[87]
5A	γ-Indomethacin	Ranitidine hydrochloride 2:1, 1:1, 1:2	Dry conditions at 4, 25, and 40 °C up to 30 days	Oscillatory ball mill	25 mL stainless steel milling jar	2 (d = 12 mm) stainless steel balls 1 g of sample per grinding cell	30 Hz	4 °C ± 2 °C	60 min	[28]
6A	γ-Indomethacin	None	Not reported	Oscillatory ball mill	25 mL stainless steel milling jar	6 (d = 9 mm) stainless steel balls 1 g of sample per grinding cell	30 Hz	4 °C ± 2 °C	6 h	[88]
	α-Indomethacin		Not reported					immersion in liquid nitrogen		
7A	Tadalafil	None	Not reported	6770 SPEX freezer/mill	Stainless steel vessel	Stainless steel rod (no balls) 1 g of sample per grinding cell	15 Hz	Cryogenic temperature (liquid nitrogen)	10 min grinding, 3 min cool-down (2 h total)	[26]
			Not reported	Planetary ball mill	250 mL zirconium jar	6 zirconia balls (d = 20 mm) 16 g of sample per grinding cell	6.6 Hz	Room temperature	15 min cycles, 5 min breaks (24 h total)	
8A	Glibenclamide	None	Not reported	6770 SPEX freezer/mill	Stainless steel vessel	Stainless steel rod (no balls) 1 g	15 Hz	Cryogenic temperature (liquid nitrogen)	6 min grinding, 3 min cool-down (3 h total)	[89]
9A	Trehalose dihydrate	None	Not reported	Spex SamplePrep 6870 freezer/mill	Polycarbonate vials (23.9 cm^3^) with steel end caps	Magnetic rod (no balls) 1 g	15 cycles per second	Cryogenic temperature (liquid nitrogen)	2 min milling, 1 min of cool-down (30 min total)	[90]
10A	Atenolol	Hydrochlorothiazide 1:1, 1:2, and 2:1	Stored in desiccators at 4 °C and 25 °C for 30 days	6770 SPEX freezer/mill	Airtight tube	1 g	10 Hz	Cryogenic temperature (liquid nitrogen)	2 min milling, 2 min cool down (48 min total)	[91]
11A	Furosemide	Tryptophan 1:1	Not reported	Oscillatory ball mill	25 mL jars	2 stainless steel balls (d = 12 mm) 500 mg	30 Hz	6 °C	90 min	[92]
	Indomethacin	Arginine								
12A	Dexamethasone	None	Not reported	High-energy planetary mill	43 cm^3^ ZrO_2_ milling jars	7 ZrO_2_ balls (d = 15 mm) 1.1 g	6.6 Hz	Room temperature	15 min milling, 5 min cool down (12 h total)	[27]
13A	α-Lactose	None	Not reported	Planetary ball mill	12 cm^3^ stainless steel jar	50 stainless steel balls (d = 5 mm) 1 g	6.6 Hz	30 ± 5% relative humidity and 22 ± 3 °C	20 min milling, 5 min cool down (1–20 h total)	[93]
14A	α-D-Glucose	None	Not reported	High-energy planetary mill	45 cm^3^ ZrO_2_ milling jar	7 ZrO_2_ balls (d = 1.5 cm) 1 g	5 Hz	−15 °C	20 min milling 10 min cool down (1 and 14 h total)	[68]
								25 °C		
15A	Mebendazole	Aspartame 1:1/1:1:1	Stored in desiccators at 40 °C and 25 °C up to 4 months	Oscillatory ball mill	25 mL ball milling jars	2 stainless steel balls (d = 12 mm) 500 mg	30 Hz	5 °C (cold room)	90 min	[94]
	Tadalafil	Phenylalanine 1:1/1:1:1								
	Piroxicam									
16A	α-D-Glucose	None	Not reported	High-energy planetary mill	45 cm^3^ ZrO_2_ milling jar	7 ZrO_2_ balls (d = 1.5 cm) 1 g	5 Hz	−15 °C	20 min milling, 10 min cool down (1, 14 h total)	[95]
	β-Glucose		Not reported					25 °C		
17A	Carvedilol	11 different amino acids 1:1	Stored at 25 °C under dry conditions for up to 2 years	Mixer mill MM400	25 mL stainless steel jars	2 stainless steel balls (d = 12 mm) 1000 mg	30 Hz	6 °C (cold room)	90 min	[31]
	Carbamazepine									
	Furosemide									
	Indomethacin									
	Mebendazole									
	Simvastatin									
18A	Salts of indomethacin	Lysine 1:1	Stored at 25 °C, and 40 °C under dry conditions up to 36 weeks	Vibrational ball mill	25 mL milling jars	2 stainless steel balls (d = 12 mm) 1000 mg	30 Hz	6 °C (cold room)	60 min	[96]
19A	Mebendazole	Tryptophan Xdrug = 0.1, 0.3, and 0.5	Not reported	Vibrational ball mill	50 mL stainless steel jars	2 stainless steel balls (d = 12 mm)	30 Hz	Room temperature	60, 120, and 150 min	[97] unpublished data
20A	18 different drugs	NaTC natural bile acid surfactant sodium taurocholate 1:1	Stored at 22 ± 2 °C	Oscillatory ball mill	25 mL stainless steel jar	1 stainless steel ball (d = 15-mm) 1 g	25 Hz	Room temperature and −10 ± 2 °C	180 min. total time, with 10 min. break every 30 min	[37]
									120 min, with 7.5 min breaks cooled in liquid nitrogen	
21A	Carbamazepine	Arginine	Not reported	Oscillatory ball mill	25 mL stainless steel jar	2 stainless steel ball (d = 12 mm) 500 mg	30 Hz	6 °C	90 min	[98]
	Indomethacin	Phenylalanine								
		Tryptophan								
22A	(S)-Naproxen	L-arginine	Stored at 25 °C, and 40 °C under dry conditions	Oscillatory ball mill	25 mL stainless steel jar	2 stainless steel ball (d = 12 mm) 1 g	30 Hz	6 °C	60 min	[99]
23A	Griseofulvin	Aspartic Ac	Stored at 23–28 °C under dry conditions up to 12 months	High-energy planetary ball mill	Stainless steel crucible	3 stainless steel balls 2.5 g	9.3 Hz	Not specified	6 h, with 0.5 min pauses every 30 min	[100]
		Lysine								
		Methionine								
		Valine								
		Tryptophan								
24A	Naproxen	Tryptophan and proline	Stored at 40 °C under dry conditions up to 332 days	Oscillatory ball mill	25 mL stainless steel jar	2 stainless steel ball (d = 12 mm) 1 g	30 Hz	6 °C	90 min	[101]
25A	Mebendazole	None	Stored at 40 °C under dry conditions up 4 weeks or 3 months	Oscillatory ball mill	25 mL stainless steel jar	2 stainless steel ball (d = 12 mm) 500 mg	30 Hz	5 °C	90–180 min	[102]
		Dipeptide 1:1								
		Aminoacid mixtures 1:1:1								
26A	Oxaprozin	RameβCD 1:1	Not reported	High-energy vibrational micro mill	Not specified	Not specified	24 Hz	Not specified	30 min	[103]
		RameβCD-Arg. 1:1:1								
27A	Furosemide	Arginine 1:1	Not reported	Vibrational ball milling	25 mL stainless steel jar	2 stainless steel ball (d = 9 mm) 500 mg	25 Hz	6 °C	99 min	[104]
	γ-Indomethacin									
	γ-Indomethacin + CA									
28A	Indomethacin	L-tryptophan 1:1	Not reported	Oscillatory ball mill	25 mL stainless steel jar	2 stainless steel ball (d = 12 mm) 1500 mg	30 Hz	6 °C	0, 5, 15, 30, 45, 60, and 90 min. 3 or 6 h	[105]
	Furosemide									
29A	Naproxen	Naproxen sodium 2:1, 1:1, and 1:2	Stored at 40 °C under dry conditions up to 2 weeks or 2 months	Oscillatory ball mill	25 mL stainless steel jar	2 stainless steel ball (d = 12 mm) 500 mg	30 Hz	4 °C	90 min	[106]
30A	Carvedilol	Glutamic Ac	Not reported	Vibrational ball mill	25 mL stainless steel jar	2 stainless steel ball (d = 12 mm) 700 mg	30 Hz	6 °C	60 min	[107]
		Aspartic Ac								
31A	Indomethacine	Arginine	Stored in refrigerator (≈5 °C)	Mixer mill MM400	25 mL stainless steel jar	2 stainless steel ball (d = 12 mm) 500 mg	30 Hz	Not specified	60 min, with 10 min pauses; cell would be in liquid nitrogen for 2 min	[36]
		Phenylalanine								
		Tryptophan								
32A	Simvastatin	Lysine	Stored in desiccators at 4 °C	Oscillatory ball mill	25 mL stainless steel jar	2 stainless steel ball (d = 15 mm) 500 mg	30 Hz	Not specified	60 min. with 10 min. pauses cell would be in liquid nitrogen for 2 min	[108]
		Serine								
	Glibenclamide	Threonine								
		Aspartic acid								
33A	Indomethacin	Arginine	Stored at 40 °C under dry conditions	Oscillatory ball mill	25 mL stainless steel jar	2 stainless steel ball (d = 12 mm) 500 mg	30 Hz	6 °C	90 min	[98]
		Tryptophan								
	Carbamazepine	Tyrosine								
		Phenylalanine								
34A	Indomethacin	Tryptophan	-	Oscillatory mill	12 mL Stainless steel jar	2 stainless steel ball (d = 10 mm) 1.2 g	10.83 Hz	Not specified	360 min	[109]
35A	Carbamazepine	Citric acid	Stored at 40 °C under dry conditions up to 2 months	Oscillatory ball mill	25 mL stainless steel jar	2 stainless steel ball (d = 12 mm) 500 mg	30 Hz	4 °C	90–180 min	[110]
36A	Arginine	Glibenclamide 1:1	Stored at 4 °C, room temperature, and 40 °C up to 13 months	Oscillatory ball mill	25 mL milling chambers	2 stainless steel balls (d = 12 mm) 500 mg	30 HZ	Not specified	60 min, chambers were cooled in liquid nitrogen	[111]
	Serine									
	Quercetin									
37A	Glutamic ac	Mebendazole 1:1 and 1:1:1	Stored at 40 °C and 25 °C in desiccators under dry conditions up to 6 months	Oscillatory ball mill	25 mL stainless steel jar	2 stainless steel ball (d = 1.2 cm) 500 mg	30 Hz	5 °C (cold room)	30, 60, and 90 min	[112]
	L-arginine									
	Glutamic Ac-Arginine									
	Arginine-glutamic ac									
	Glutamic-arginine									
38A	Mefenamic acid	Meglumine 1:1, 1:2, and 1:4	Not reported	Planetary ball mill	Not specified	5 stainless steel balls (d = 10 mm)	4.16 Hz	Not specified	20 min	[113]
	Indomethacin	PVP 1:1, 1:2, and 1:4								
39A	L-methionine	Rutin 1:1, 1:2, 2:1	Not reported	Planetary ball mill	45 mL zirconia jar	8 YTZ balls (d = 10 mm)	10 Hz	Room temperature	12 h with a break every 10 min	[114]
	Naringin hydrate									
	Quercetin dihydrate									
	Hesperidin Chlorothiazide Indapamide Triamterene Nifedipine									
40A	Benzamidine	Gliclazide 1:1, 1:5, or 5:1	Stored in a desiccator at 22 ± 2 °C, and 40 °C under relative humidity up to 180 days	Oscillatory ball mill	25 mL stainless steel milling jar	Stainless steel ball (d = 15 mm) 0.25 g	25 Hz	Cromilling inmersing jars in liquid nitrogen for 5 min prior to milling. 7.5 min milling	180 min, with a cool down period of 15 min after every 30 min	[38]
41A	Arginine	Quercetin 1:1, 1:2	Not reported	Not specified	25 mL stainless steel	1–3 stainless steel ball (d = 18, 15, and 12 mm)	Not specified	2 h	Not specified	[115]
	Glutamic acid									
	Aspartic acid									
	Tryptophan									
	Glycin									
42A	Candesartan cilexetil	Hydrochlorothiazide	Stored at 4 °C, 30 °C, and 40 °C under dry conditions up to 90 days	Planetary ball mill	125 mL stainless steel grinding jars	3 stainless steel grinding balls (d = 10-mm) 2 g	9.3 Hz	Room temperature	2.5 h	[116]
		Hydroxypropyl methylcellulose								
		Acetate succinate (HPMCAS) type M								

**Table 2:** Conditions of preparation of co-crystals by grinding method.

#	Sample	Molar Ratio	Method of Preparation	Milling Type	Instrument Brand	Milling Jar	Balls (# and Material)	Milling Frequency	Milling Temp	Milling Time	Ref.
1C	Nicotinamide: L-(+)-Ascorbic acid	1:1	Assisted by solvent	Vibrational	Mixer Mill (IST 500) InSolido Technologies	Polymethylmetacrylate	Two stainless steel balls	30 Hz	NR	60 min	[66]
2C	Salicylic acid:2-pyridone Salicylic acid: 4-Pyridone	1:1	NR	Vibrational	Mixer Mill (IST 500) InSolido Technologies	Polymethylmetacrylate	Two stainless steel balls	30 Hz	NR	50 min	[117]
3C	Ciprofloxacin- thymol	1:2	Assisted by solvent (EtOH)	NR	Retsch MM200 ball miller,	NR	NR	20 Hz	NR	30 min	[118]
4C	Urea- caffeine	1:1	NR	Oscillatory ball	Mixer Mill MM400-Retsch GmbH, Haan	Stainless steel jar	One 15 mm stainless steel ball	25 Hz	Room temperature	60 min	[119]
5C	Brexpiprazol-Catechol Brexpiprazol-Succinic acid	1:1	NR	NR	Nano Ball Mill (Fritsch Premium Line, FRITSCH GmbH, Idar-Oberstein, Germany) using	NR	Stainless steel balls	8.3 Hz	NR	120 min	[120]
6C	Quercetin- malonic acid	1:1 and 1:2	Solvent drop grinding	NR	NR	NR	NR	NR	NR	30 min	[121]
7C	Paracetamol-trimethylglycine	1:1	NA	Planetary ball	QM-3SP2, Nanjing NTU Instrument Co.	NR	NR	6.6 Hz	NR	5 h	[44]
8C	Meloxicam- benzoic acid	1:1	LAG	NR	Retsch CryoMill	NR	NR	25 Hz	Room temperature	30 min	[122]
9C	Acetazolamide and 4-hydroxybenzoic acid	1:1	LAG	Planetary ball	QM-3SP04, gear type	25 mL stainless steel milling jars	NR	25 Hz	NR	30 min	[123]
10C	Furosemide-urea and carbamazepine-indomethacin	1:1	LAG	NR	Retsch MM400 ball mill	50 mL jar, with two 5 mm stainless steel balls and drops of acetone.	NR	NR	NR	60 min	[51]
11C	Ciprofloxacin-nicotinic and isonicotinic acids	1:1	Assisted or not by solvent (EtOH)	NR	Retsch MM 400 mixer mill	10 mL stainless-steel jars	1 stainless steel ball of 7 mm diameter, 100, 500 mg sample	30 and 15 Hz	NR	30 min	[124]
12C	Pyrazinamide-diflunisal	1:1	LAG	Oscillatory ball mill	Mixer Mill MM400	25 mL stainless steel milling jars	NR	15 Hz	Room temperature	60 min	[125]
13C	Acetazolamide–4-aminobenzoic acid	1:1	With solvent	Planetary ball	Fritsch micro mill model Pulverisette 7	12 mL agate grinding jars	Ten 5 mm agate balls	8.3 Hz	NR	30 min	[67]
14C	Acetazolamide-nicotinamide-2-pyridone	1:1:1	LAG with ethyl acetate and tetrahydrofuran solvents	Planetary ball	QM-3SP04, gear type	25 mL stainless steel milling jars	NR	15 Hz	NR	60 min	[126]
15C	β-Lapachone-resorcinol	1:1	LAG	NR	Retsh Mixer Mill (Model MW 200)	Stainless steel jar together	A stainless steel ball	20 Hz	NR	20 min	[127]
16C	Norfloxacin-nicotinic acid	NR	NT and LAG	Ocillatory ball system	Mixer Mill MM 400, Retsch GmbH and Co	Stainless steel jars	7 mm diameter stainless steel ball	15 Hz	NR	30 min	[128]
17C	Chlorothiazide, D-proline, L-proline	1:1	NT and LAG	Oscillatory ball	Retsch (MM400, Retsch)	NR	NR	30 Hz	NR	30 min	[129]
18C	Praziquantel, poloxamer F-127, and sucrose stearate	20:1, 10:1, 10:2, and 10:3	NT	High-energy vibrational ball	Mixer Mill MM 200, Retch, GmbH	10 mL volume stainless steel grinding jars	Two 7 mm stainless steel grinding balls	25 Hz	28.10–30.34 °C	30 or 90 min	[130]
19C	Ferulic acid, urea, nicotinamide, and isonicotinamide (INA)	1:1 and 1:2	LAG	NR	Retsch Mixer Mill (model MM301)	Stainless steel grinding jar	One 7 mm stainless steel ball	20 Hz	NR	20 min	[131]
20C	Ketoconazole, fumaric acid, and succinic acid	1:1.1 and 1:1	NT and LAG	Oscillatory ball	Retsch MM 400	25 mL stainless steel jars	One stainless steel ball	19 Hz	NR	60 min	[132]
21C	Itraconazole: 4-aminobenzoic acid Itraconazole: 4-hydroxybenzamide	1:1 2:1 1:2	LAG	Planetary micro	Fritsch planetary micro mill, Pulverisette 7	12 mL agate grinding jars	Ten 5 mm agate balls	8.3 Hz	NR	40 min	[133]
22C	S-ibuprofen: nicotinamide	1:1	N.R	Oscillatory ball	MM400—Retsch	10 mL ZrO_2_ milling jars	One ball, 10 mm	30 Hz	NR	60 and 10 min and 5 min pauses	[134]
23C	Pyrazinamide: 4-aminosalicylic acid	1:1	LAG	Planetary ball	QM3SP04, gear type, Nanjing University Instrument Factory	20 mL stainless steel grinding tank	N.R	20 Hz	Room temperature	40 min	[135]
24C	Theophylline: 4-aminobenzoic acid	1:1	N.R	N.R	MM 400, Retsch, Germany	10 mL jar 25 mL jar	One ball, 8.74 mm, One ball, 13.72 mm	30 Hz	N.R	Period times: 2,5,10, 15, 20, and 25 min	[136]
25C	Betulin-terephthalic acid	1:1 2:1	Assisted by solvent	NR	SPEX 8000 mixer mill (CertiPrep Inc., Metuchen, NJ, USA)	60 mL steel jar	Steel balls 6 mm	NR	NR	Pre-milled: 5 min After solvent: 10 min	[137]
26C	5-Fluorocytosine:5-fluorouracil	1:1	NT SDG	Oscillatory	Mixer Mill MM400 RETSCH	25-mL stainless steel milling jar	Two 7 mm stainless steel balls	25 Hz	Room temperature	90 min SDG: 60 min	[138]
27C	Nicotinamide:adipic acid (polymorph, form 2)	1:1	Assisted by solvent (acetonitrile)	NR	Retsch MM400 mill (in-house modified)	Stainless steel milling jar	Two 7 mm stainless steel balls	30 Hz	NR	60–90 min	[139]

LAG: liquid assisted grinding; NT: neat grinding, SDG: solvent drop-grinding; NR: not reported.

**Table 3:** Conditions of preparation of polymorphs by mechanical activation.

#	Sample	Obtained Polymorph	Mill Type	Milling Cell	Ball (#, Material) Sample Weight	Milling Frequency	Milling Temperature	Milling Time and Solvent	Ref.
1P	Ranitidine hydrochloride	Ranitidine hydrochloride, form 2	Oscillatory ball mill (mixer mill MM301, Retsch GmbH and Co., Weinheim, Germany)	25 mL Stainless steel	2 stainless steel balls (d = 12 mm) 1 g s	30 Hz	12 ± 3 °C	180 min, stop every 30 min to scrape and remix powder	[74]
		Ranitidine, form 2 (with traces of form 1)					35 °C	120 min, stop every 30 min to scrape and remix powder	
		Ranitidine, form 2						240 min, stop every 30 min to scrape and remix powder	
2P	Chlorhexidine dihydrochloride	2-step polymorphism produces ChxHC form 2 as a precursor of form 3	High-energy planetary mill (Pulverisette 7; Fritsch, Idar-Oberstein)	43 cm^3^ ZrO_2_	7 ZrO_2_ balls (d = 15 mm) 1 g	6.6 Hz	Room temperature	12 h (15 min milling periods with 5 min rests)	[140]
3P	Γ-sorbitol	A form sorbitol	High-energy planetary micro-mill (Pulverisette 7; Fritsch, Idar-Oberstein)	45 cm^3^ zirconium	7 zirconium balls (d = 15 mm) 1 g of sample	6.6 Hz	Room temperature	10 h	[34]
4P	Rivastigmine (RHT form 2)	RHT form I	Retsch planetary ball mill PM100	50 mL stainless steel	3 stainless steel balls (d = 20 mm) 1 g	6.6 Hz	Room temperature	3 h (stopping at 15 min, 30 min, 1 h and 2 h)	[141]
5P	o-Aminobenzoic acid (mixture of FII and FIII forms)	FIII form	Oscillatory ball mill (Mixer mill MM400, Retsch GmbH and Co., Germany)	25 mL stainless steel	One stainless steel ball (d = 15 mm) 0.5 g 30 μL of solvent	25 Hz	Room temperature	2.5 h (30 min milling periods with 15 min pauses) Solvent: valeric acid (FIV and FIII)	[54]
		FII form							
	m-Aminobenzoic acid (FIII form)	FIV form							
		FIV and FIII							
	Carbamazepine	FIV form							
	p-aminobenzoic acid	β-PABA			1 stainless steel ball (d = 15 mm) 0.5 g 30 μL of solvent		Cryogenic temperature (immersed in liquid N_2_ for 5 min prior to miling every 7.5 min)	2.5 h (7.5 min milling and 2.5 min pauses in liquid nitrogen) Solvent: valeric acid, 10% acetamide or ethanol. (FI)	
	o-Aminobenzoic acid (mixture of FII and FIII forms)	FI form (FII converts to FIII and subsequently FIII converts to FI.)							
		FI form							
6P	Dexamethasone	DEX form A and B	High-energy planetary mill (Pulverisette 7, Fritsch, Idar-Oberstein)	43 cm^3^ ZrO_2_	7 ZrO_2_ balls (d = 15 mm) 1.1 g	6.6 Hz	Room temperature	12 h (15 min milling periods, with 5 min rests)	[27]
7P	Sofosbuvir (anhydrous form 1)	Form A or B	Vibrational ball mill (MM400, RETSCH)	5 mL stainless steel	2 stainless steel balls (d = 5 mm) 50 mg 10 μL of Solvent	25 Hz	Room temperature	30 min Solvent: water or methanol	[79]
		Form A						30 min Solvent: anisole, n-butyl acetate, or ethyl acetate	
		Form A (form 1 changes to form V)						30 min Solvent: anisole	
		Form A						60 min, solvent: tetrahydrofuran	
		Form A (form 1 changes into form B and then forms A)						20 min, solvent: butyl acetate or ethyl acetate	
8P	Sulindac (form II)	Form II and form I	High-energy planetary mill (Pulverisette 7eFritsch)	43 cm^3^ ZrO_2_	7 ZrO_2_ balls (d = 15 mm) 1 g	6.6 Hz	Room temperature	5 min	[69]
		Form I						600 min (10 min milling, with 5 min pauses)	
		Mixture of form II and form I						20 min (10 min milling periods, with 5 min pauses)	
9P	Γ-sorbitol	A form sorbitol	High-energy planetary mill (Pulverisette 7-Fritsch)	43 cm^3^ ZrO_2_	7 ZrO_2_ balls (d = 15 mm)	6.6 Hz	Room temperature (dry nitrogen atmosphere)	10 h	[75]
	Mannitol (β)	α Mannitol							
	Mannitol (δ)	α Mannitol							
10P	Famotidine (form B)	Form A (form B to A transformation ratio increased with milling time)	Oscillatory ball mill (Mixer Mill MM301, Retsch GmbH and Co., Germany)	25 mL stainless steel	2 stainless steel balls (d = 12 mm) 0.2 g	15 Hz	130 °C	10 min	[142]
							110 °C	20 min	
							110 °C	30 min	
11P	Gabapentin (GBP) form I	GBP form II	Oscillatory ball mill (Mixer Mill MM301, Retsch GmbH and Co., Germany)	25 mL stainless steel	2 stainless steel balls (d = 15 mm) 0.2 g of sample	20 Hz	Room temperature	120 min	[76]
	GBP form II	GBP form III						105 min	
		GBP form IV						120 min	
	GBP form III	GBP form II						15 min	
		GBP form III (produced by the coexistence of form I and II after 15 min milling)						60 min	
		GBP form IV						105 min	
	GBP form IV	GBP form II						2 min	
		GBP form III						30 min	
		GBP form IV						105 min	
12P	Ciprofloxacin salicylate (monohydrate)	Form I (after 4 min of neat grinding) From 2 (after 9.5 min of neat grinding)	Fritsch planetary micro mill, model Pulverisette 7	12 mL agate	10 agate balls (d = 5 mm) 0.1 g 60 μL of solvent	8.3 Hz	NR	50 min, solvent: water, and the use of water/organic solvents decreases the time of existence for form I	[143]
	Ciprofloxacin salicylate (3.67 hydrate)	Form II (after 17 min of neat grinding)							
	Anhydrous ciprofloxacin salicylate	From I							
13P	γ-sorbitol	Form α (complete transformation)	High-energy planetary mill (Pulveri- sette, 7-Fritsch)	43 cm^3^ ZrO_2_	7 ZrO_2_ balls (d = 15 mm)	6.6 Hz	Room temperature	180 min (10 min milling periods, with 5 min rests)	[144]
14P	Ethenzamide: ethylmalonic acid (Co-crystal)	Form l (SDG with n-hexane) Form ll (after neat grinding or SDG with toluene or cyclohexane)	Oscillatory ball mill (Mixer Mill MM301, Retsch GmbH and Co., Germany)	10 mL stainless steel	1 stainless steel ball (d = 7 mm) 0.1 g of EA and 0.0799 g of EMA (1:1 molar ratio) 0.05 mL of solvent	20 Hz	Room temperature	15 min, solvent: toluene, cyclohexane, or n-hexane	[145]
15P	Caffeine: glutaric acid (co-crystal)	Form l (after neat grinding and SDG with n-hexane, cyclohexane or heptane)	Oscillatory ball mill (Mixer Mill, Retsch GmbH and Co., Germany)	Stainless steel (volume NR)	2 stainless stell balls (d = NR) 0.75 g (1:1 molar ratio)	30 Hz	Room temperature	60 min Solvent: n-hexane, cyclohexane, or heptane	[146]

NR: not reported; SDG: solvent drop grinding.

## 4. Evaluation of Physicochemical Properties of Co-Amorphous, Co-Crystals, and Polymorphs Induced by Mechanical Activation

With the purpose of evaluating the outcomes of the milling process, different characterization techniques are applied to determine structural changes and their effects on the properties of the final pharmaceutical formulation. This section is divided into solubility evaluation, intermolecular interactions by spectroscopic techniques, such as Raman, Infrared, and ss-NMR, phase transitions by thermal analysis techniques, and structural characterization by X-ray diffraction. An overview of results for each kind of drug formulation (amorphous, co-crystal, or polymorph) is presented for each characterization technique. An additional section on characterization techniques by microscopy is included. This last section refers to the methods that have been used little, until the moment of elaboration of this review but that provide relevant information, regarding the formulation’s characteristics.

### 4.1. Evaluation of Solubility Enhancements as an Effect of the Milling Process

Solubility enhancement is an essential property for developing novel drugs. Solubility evaluation results may be expressed in different ways, for example, powder dissolution and intrinsic dissolution rate (IDR); however, both studies compare the solubility enhancement of the crystalline materials and formulation after milling. In the case of powder dissolution, analyses are performed using only the systems in powder. In contrast, the intrinsic dissolution rate (IDR) can be defined as the dissolution of a drug substance under specific conditions, such as a constant surface area and agitation speed [91].

Table 4 and Table 5 provide an overview of the solubility results reported for amorphous, co-amorphous, and co-crystals. As mentioned before, in the first column of the tables, a code with a number and letter is used to identify each drug formulation. In each code, the letter stands for the following criteria: A—amorphous, C—co-crystal, and P—polymorph. Note that in Table 4, Table 5 and Table 6, the codes in the column are not consecutive numbers because not all articles analyzed their formulations with all the characterization techniques. Therefore, data are only exhibited in the tables when the articles performed those studies. All the articles report solubility enhancements in diverse ways, such as folds, solubility value, or dissolution rate, using various units. The articles that did not report folds have been marked with an asterisk (*); to simplify the analysis, those values were converted to folds using the formula:

(1) $$\mathrm{Folds}\;\mathrm{Increase}=\frac{\mathrm{Increased}\;\mathrm{solubility}\;\mathrm{value}}{\mathrm{Solubility}\;\mathrm{value}\;\mathrm{of}\;\mathrm{crystalline}\;\mathrm{or}\;\mathrm{unprocessed}\;\mathrm{material}}$$

It is important to mention that no information of solubility regarding polymorphs (obtained by milling) was found.

(a) Solubility for co-amorphous systems after ball milling

As seen in Table 4, it is relevant to note that a constant dissolution rate verifies that the drug in the co-milled sample does not recrystallize during dissolution. The steady behavior shows that the interaction between two drugs or drug–excipient in the amorphous binary system is strong and stable enough to prevent structural rearrangement during dissolution. Moreover, extended times in intrinsic dissolution studies (where no changes in rate are observed) show that bioavailability would not be decreased due to recrystallization in in vivo conditions [87]. Except from the LAG sample reported by Kasten et al. [96], the articles typically show a decrease in dissolution rate.

There are many co-amorphous formulations prepared by milling, in which acidic and basic excipients were used to form salts. The article that shows the highest increase in solubility was published by Kasten et al. [31], using both DBM and LAG as preparation methods. They found that the co-amorphous salt formulations of basic AAs and acidic drugs had the most significant increase in dissolution rate. The use of amino acids, particularly arginine (a basic amino acid)-based salts, showed substantial dissolution enhancement, combined with acid drugs, approximately 140–431.8-fold, when compared to the amorphous drug, possibly due to strong molecular interactions attributed to salt formation. Therefore, the salt formation of an acid-basic system could be a meaningful approach to enhancing solubility properties in drug formulations. Other milling conditions were also analyzed for amorphs and co-crystals to determine if milling conditions directly affect the solubility of the obtained system. Apparently, long milling times do not affect the increase of solubility. Caron et al. [86] measured 15 h, in total, of effective milling, and sulfadimidine-polyvinylpyrrolidone had an increase of 26.5 times its solubility. Whereas Kasten et al. [31] milled a wide variety of samples for a total of 90 min and showed that increases in solubility ranged from 0.9 to 431.8 times.

For co-amorphous, milling time is relevant to obtaining the new drug formulation; nevertheless, once amorphization is achieved, longer milling times do not enhance solubility. This demonstrates that properties and possible interactions between drug–drug or drug–excipient are more important than long milling times to increase solubility. Finally, in Table 4, no trend is observed, regarding the type of mill or milling cell material towards affecting solubility enhancement. These milling conditions are relevant for the obtention of the amorphous and co-amorphous systems. Still, they do not seem to have an impact on the increase of the solubility of the sample. There is a possibility that 30 Hz might be the optimal milling frequency, as the highest increase in solubility was observed at this speed (at 1:1 molar ratio), but it should also be noticed that all these articles [31,85,94,96,102] used amino acids for the experiments, which could be a relevant factor influencing the solubility.

(b) Solubility of co-crystals after grinding

Comparing results from Table 4 and Table 5, the co-crystals’ primary preparation method is solvent-assisted, and solubility enhancement ranges from less than 1-fold to a maximum of 20 times. The works of Arabiani et al. [120] and Zhao et al. [44] have shown that it is possible to obtain co-crystals under dry conditions. Still, solubility was respectively little (1.056-fold) or not enhanced at all (0.86-fold, compared to paracetamol alone) (see Table 5). On the other hand, independently of the API, studies with amorphous systems clearly show a higher increase in solubility than co-crystals, as shown in Table 4 and Table 5. Several authors have suggested that the physicochemical properties (melting temperature, solvation, etc.) of all the components of the co-crystal, as well as the solution properties of the medium (pH, surfactant, etc.), can significantly influence the solubility and dissolution of the co-crystals [127,147,148]. Other authors have mentioned that this induced improvement in solubility could possibly be the effect of the co-former being drawn out of the crystal lattice and into the aqueous medium [149]. For hydrophilic co-formers of co-crystals [121,124] interactions might be developed with -OH groups from water molecules by new hydrogen bonding, resulting in an enhancement of drug solubility. This theory is valid for a hydrophilic co-formers [44,127]; however, depending on the properties of the co-former, other factors, such as pH, could be more suitable to increase solubility, such as low pH for acid co-formers [124]. To sum up, it is necessary to release co-crystals in a suitable medium to improve dissolution behavior.

The results are similar to co-amorphous, in terms of the milling conditions to obtain co-crystals. As mentioned before, long milling times do not affect the increase of solubility. In fact, the longest milling time was performed by Zhao et al. [44] under dry conditions of paracetamol-trimethylglycine, and the solubility of the ball-milled co-crystals turned out to be lower than the paracetamol alone; the authors argue that supramolecular interactions, such as hydrogen bonding, might have caused this decrease in solubility. Anyway, only Shemchuk et al. [118] and Setyawan et al. [121] performed solubility studies at molar ratios different than 1:1. Still, no relation was observed to conclude that a specific molar ratio might render a higher increase in solubility. As previously mentioned for amorphs, in Table 5, no trend is observed regarding the type of mill, milling cell material, or milling speed towards affecting solubility enhancement.

To the authors’ knowledge, the solubility of polymorphs has not been studied in vitro or in vivo. Still, it would be worth analyzing whether there are significant differences in solubility between one form and the other, as one form of the crystalline drug could show better properties and, therefore, novel applications for therapeutics. A parameter related to improving properties, such as solubility or stability of a system, is the formation of the interaction between the formulation components. Therefore, the most widely used techniques for structurally analyzing co-amorphous, co-crystal, or polymorphous systems will be described then.

### 4.2. FT-IR Spectroscopic Evaluation of Intermolecular Interactions Induced by Ball Milling

Fourier transform infrared spectroscopy (FT-IR), Raman, and solid-state nuclear magnetic resonance (ss-NMR) are the primary intramolecular methods of probing the sample at the molecular level [16]. Table 6, Table 7 and Table 8 show an overview of the main spectroscopic results (FT-IR, DRIFTS, ATR-FT-IR Raman, and ss-NMR) reported to identify and study the structural rearrangement and possibility of recognizing new interactions in the formulation. Changes in the spectra from the initial crystalline materials to another form of the drug formulation (call it amorphous or co-amorphous system, co-crystal, or polymorph) might be expressed in different forms, such as peak formation, reduction of signal, the disappearance of peaks, and the merging of bands. The overall changes in each drug formulation will be explained in detail in the following subsections. Table 6, Table 7 and Table 8 show the analytical technique used, characteristic signals, and interpretation of each API change.

(c) Structural characterization of amorphous systems by spectroscopy techniques

Among the articles analyzed for amorphous and co-amorphous systems, the technique mainly used for spectroscopic characterization is FT-IR and Raman. For the infrared spectroscopy results, band shifting indicates that the system is suffering changes in the internal structure. It is important to notice is that a relation between the shifts and hydrogen bonding has been found, as shifts towards a higher wave number may be linked to the loss of hydrogen bonds [24], while a shift to a lower wavenumber is related to the formation of hydrogen bonding. A more stable amorphous state would be expected [97].

In the case of studies that performed Raman spectroscopy, all of them reported shifts in the spectra or band broadening, which conclude the possible formation of interactions between the components at a molecular level. It is essential to mention that both bathochromic and hypsochromic shifts happen due to variations in molecular conformation and intermolecular bonding of amorphous forms [88]. Due to the fact that Raman is not affected by the polarizability of water molecules, another meaningful use of this technique, along with UV imaging, is to study dissolution behavior, as it reveals potential changes in the physicochemical properties of the crystalline and amorphous drugs, as well as solid-state changes during dissolution; case in point, the co-amorphous systems prepared by Ueda et al. showed changes in the spectra of the samples, which were clear indicators of recrystallization [106]. Finally, from all the papers analyzed, it was observed that another application of Raman is to quantify the amorphous content of a drug as milling time increases; this is called apparent amorphicity (%) and has been studied to observe rising levels of amorphizing material [93,150].

Finally, in Table 6, the usefulness of NMR in amorphous systems is that it gives information regarding the thermal degradation of samples after milling. For example, Oliveira et al. [27] concluded during their study that the NMR spectrum of the milled dexamethasone was totally similar to that of the initial one, as it showed that a high-energy mechanical action is capable of amorphizing the sample without inducing chemical degradation, contrary to the spectra obtained from melt quenching, where the method of preparation may cause degradation.

(d) Structural characterization of co-crystals by spectroscopy techniques

FT-IR and Raman are the analytical techniques commonly used for co-crystal identification. As can be observed in Table 7, Raman spectroscopy is an advantageous technique for the analysis of co-crystals, particularly when the samples are hydrated because monitoring of water presents low Raman scattering [151], in comparison to FT-IR, which can have an uptake of humidity from the air and show the presence of a broad -OH band. Analysis from Table 7 shows that FT-IR does not seem to be the most common technique for interpreting co-crystal formation prepared by ball milling. However, there are some studies where FT-IR has been successfully used for identifying co-crystals [152,153]. In these cases, co-crystals were prepared by methods other than grinding, such as solvent evaporation or sublimation.

In Raman, it has been suggested that the shift in the conformer to lower or higher wavenumbers with the corresponding reduction in the band intensities affect the distribution of the electron cloud and suggests the formation of a co-crystal and not simply a physical mixture [44]. Several studies argue that the spectra confirm the effect of hydrogen bonding interaction in the complex formed, which is key to co-formation, rather than a simple mixture of the two starting reactants [123].

A study performed by Elsei et al. [140] supports the idea of Oliveira et al. (mentioned in the spectroscopic techniques for amorphs section)—that when no changes are observed between the ^1^H NMR milled and non-milled spectra, it allows for confirmation that the samples can be safely ball-milled without inducing thermal degradation, compared to other techniques, such as melt quenching. This has been confirmed by ^1^H NMR, ^13^C, and ^15^N spectroscopy [154].

(e) Spectroscopic studies reported for polymorphs obtained by ball milling

Table 8 summarizes several authors’ interpretations, regarding the analysis of polymorphic transformations by spectroscopic techniques. During mechanochemical milling, certain forms of drugs can be produced; however, due to the low glass transition temperature of the drug (further discussed in the phase transition by thermal techniques section), they are not necessarily stable, which results in reversion into a more stable crystalline form. Therefore, identifying polymorphs is imperative for formulation developments and complying with the regulatory authorities [141]. As shown in Table 8, each polymorph of a drug exhibits specific bands that allow a clear identification in FT-IR and Raman. After polymorphic transformation, some bands may disappear (due to conversion from one form to another), and new peaks with increased intensity now show up, thus allowing for the identification of the new polymorph. Less common, but also seen, is the shift of bands, which also indicates polymorphism. Finally, regarding polymorphism, an example is presented here to make this section clearer: in the spectra of a ball-milled sample that shows peaks from two different forms, form A and form B, this would be an indicator that the mixture contains both polymorphs; this indicates that more milling time is necessary to reach full conversion into a specific form (from A → B or vice-versa), where only the peaks of one specific form will be noticeable.

ssNMR has been little used, but it is useful to observe that the disappearance of bands indicates a change in conformational properties, such as the arrangement of molecules in the unit cell and coarsening process [27]. The ^1^H NMR proton spin-lattice relaxation time measured at various temperatures may be used to differentiate between various polymorphic forms of a drug [155].

Contrary to amorphous systems and co-crystals, to the author’s knowledge, ^1^H NMR cannot be used in these cases to observe if the polymorph suffers thermal degradation, because proton NMR signals change as a new polymorphic form develop, but further investigation needs to be performed in this field.

### 4.3. Thermal Analysis Techniques to Study Phase Transitions Induced by Grinding

Regarding the thermal analysis of samples, the most commonly used technique reported for the study of milled formulations is differential scanning calorimetry (DSC). This technique identifies phase transitions as a function of a heating process (melting, crystallization, decomposition, and glass transition temperatures). Another technique is thermogravimetry (TGA), which measures the loss of mass as a function of the temperature, due to loss of water [44] or volatile samples [124], respectively. The most common rate used is 10 °C/min, but the smaller heating ramps of 5 °C/min [68,95,100] and 2 °C/min in several articles have also been used (see Table 9). It is well-known that many transitions, such as crystallization, decomposition, evaporation, etc., are kinetic events, as functions of time and temperature. Therefore, the transition will shift to a higher temperature when heated at a higher rate. Another transition that can also be affected by the heating speed is the glass transition temperature; its shift is the result of some events. First, the temperature of the center of the sample lags the temperature of the surface. The temperature lag increases with the heating rate and causes the glass transition to shift to a slightly higher temperature. Secondly, the glass transition is associated with a change in molecular mobility, and this mobility has a small time-dependent or kinetic contribution [156].

Table 9, Table 10 and Table 11 show all the thermal characterization and phase transitions of co-amorphous, co-crystals, and polymorphs. The following sections discuss specific results for each kind of formulation.

(f) Thermal analysis of ball-milled co-amorphous systems

After analyzing the thermal characterization results of the amorphous and co-amorphous samples obtained by milling (shown in Table 9), it can be concluded that the determination of glass transition temperature (Tg) is a very useful tool to reach conclusions of amorphization of the material. For binary systems, detecting a single Tg is a clear indication of a homogeneous, single-phase, co-amorphous mixture [94]. Most of the co-amorphous system reported a single Tg, except Wu et al. [102], who prepared a total of nine co-amorphous systems and found two Tgs in the mebendazole-histidine-glycine ternary system; the rest showed only one Tg.

Several articles report the values of Tg at different molar ratios, namely 1:1, 1:2, and 2:1. In some cases, the determination of Tg is not possible, due to fast recrystallization or because it is not reported, but the rest of the articles reported the value of Tg at each molar ratio. In most cases, Tg’s value at 1:1 ratio tends to be between the values at ratios of 1:2 and 2:1. When the composition is different than 1:1, the newly observed Tg tends to be closer to the Tg of the component present in excess within the mix [87,157]. This is because the excess components in a mixture show a tendency to recrystallize [158]. These shifts in the value of Tg give clear information regarding the development of new interactions of the components in the sample, and this is where the Gordon–Taylor equation is very relevant. The theoretical Tg for a co-amorphous system containing two amorphous components can be calculated with this equation [159]

(2) $$T_{g1,2}=\frac{w_{1}T_{g1}+{\mathrm{Kw}}_{2}T_{g2}}{w_{1}+{\;\mathrm{Kw}}_{2}}$$

where Tg_1,2_ is the glass transition temperature of the co-amorphous mixture, w_1_, w_2_, Tg_1_, and Tg_2_ are the weight fractions and glass transition temperatures for the two amorphous components, and K is a constant expressed as:

(3) $$\;K=\frac{T_{g1\;}\times \rho_{g1}}{T_{g2\;}\times \rho_{g2}}$$

where ρ_1_ and ρ_2_ are the densities of each of the two components [92].

The Gordon–Taylor equation assumes no interaction between the molecules in the mixture; therefore, large deviations could suggest that the two components interact at the molecular level [87]. A negative deviation from the predicted value of Tg by the Gordon–Taylor equation indicates a non-ideal mixing [158,160,161]. In this sense, free volume additivity, interactions between components, and loss of hydrogen bonding during mixing could account for this non-ideal mixing and negative deviations [160]. On the other hand, it has been mentioned that, when the Tgs of the co-amorphous systems are higher than the Tgs (a positive deviation) calculated by the Gordon–Taylor equation, it suggests strong molecular interactions between the components [92,96]; such interactions can be hydrogen bonding [162], π–π interactions [98], and salt formation [163] between the drug and co-former, thus leading, again, to a rise in the value of the experimental Tg over the theoretical Tg [94]. This deviation between theoretical and experimental Tg strongly depends on the drug–drug or drug–co-former selected for study. It is worth mentioning that Kasten et al. [31] concluded that the highest increase in Tgs occurred in the acidic drug basic AAs combinations (See Table 9), due to interactions resulting in salt formation. As was mentioned in Section 3.2, amorphization for milling requires to be performed at temperatures far below from the glass transition temperature; as shown in the data from Table 9, all reported experimental conditions agreed with this statement.

(g) Phase transitions reported for co-crystals prepared by milling

After analyzing the data presented in Table 10, it was concluded that DSC can identify the melting point of co-crystals, as it is, in general, remarkably different from the pure melting temperatures of APIs and pure co-former [44]. Identifying new endothermic peaks between the melting points of both components indicates the formation of the co-crystal phase [121,124,127].

**Figure 2:**
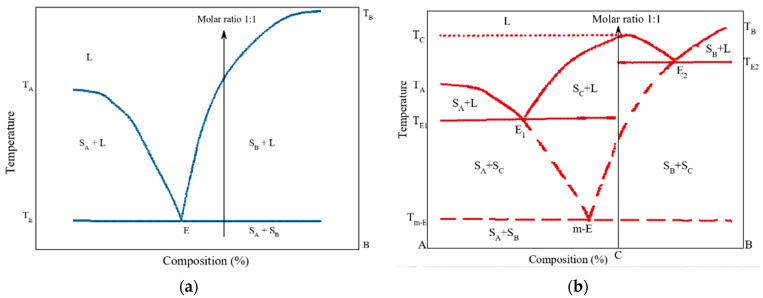
(**a**) Binary phase diagram of a combination incapable of co-crystal formation. (**b**) Binary phase diagrams of co-crystal formation. L, liquid; S_A,_ solid of component A; S_B_, solid of component B; T_E_, eutectic temperature; S_C_, co-crystal; E, eutectic point; m-E, metastable eutectic point; T_m-E_ metastable eutectic temperature; T_A_, melting temperature of component A; T_B_, melting temperature of component B; T_C_, melting temperature of co-crystal. Obtained and replotted from [71,72].

According to Stoler et al. [70], identifying a eutectic mixture in a phase diagram will result in a classic V shape (where the minimum point represents the eutectic point). By contrast, the binary-phase diagram for a co-crystal exhibits two eutectic points and a region of co-crystal at the maximum between the two eutectic points, resulting in a W-shaped phase diagram for co-crystals [71,72,164] (See Figure 2 for a representation of these diagrams).

In conclusion, for co-crystals ball-milled samples, endothermic peaks usually are located between the melting points of the parent compounds to proof the co-crystal formation (See Table 10); except, Nugrahani et al. [165] and Macfhionnghaile et al. [119] found values of Tm of the co-crystal lower than the parent drug, and Zhao et al. [44] found two endothermic peaks in the sample analyzed.

(h) Phase transitions of polymorphs resulting from mechanical activation

After reviewing the results of the thermal analysis presented in Table 11, it can be concluded that DSC is a valuable technique to identify phase transitions. With DSC, it is also possible to observe reminiscence of residual solvents [79] and melting temperature (Tm) to identify polymorphs. Between two polymorphs, a higher melting point would indicate a more stable form of the drug.

Other transitions, such as crystallization temperature (Tc) and other endothermic signals, are also reported (along with the articles) and summarized in Table 11. For example, Elisei et al. (Elisei et al., 2018) determined two different crystallization temperatures, one for form 2 (Tc = 124 °C) and another for form 3 (Tc = 157 °C). Finally, a melting temperature of form 3 (Tm = 256 °C) from chlorohexidine dihydrochloride polymorph. In conclusion, endothermic peaks, such as melting temperatures, are very important because higher values lead to more stable polymorphic forms, and lower values lead to metastable forms.

As mentioned in Section 3.2, crystallization and polymorphic transformations occurred during the milling process at temperatures above the glass transition temperatures; however, most of the studies of co-crystals or polymorphs do not report Tg values of the materials.

**Table 11:** Overview of thermal characterization (DSC) of drug polymorphs obtained by ball milling.

#	Sample	Polymorph Identified	Transition Temperature (°C)	Milling Temperature	Conditions and Milling Time	Ref.
1P	Ranitidine hydrochloride	Form 1	Tm = 142.73	12 ± 3 °C and 35 °C	0 to 160 °C, 10 K/min	[74]
		Form 2	Tm = 145.01			
2P	Chlorhexidine dihydrochloride	Form 2	Tc_2_ = 124	Room temperature	5 °C/min	[140]
		Form 3	Tc_3_ = 157			
		Form 3	Tm_3_ = 256			
3P	Γ-sorbitol	Form A	Decrease in melting temperature (value not reported)	Room temperature	NR	[34]
4P	Rivastigmine (RHT form II)	Form II	Tm_1_ = 97.5, Tm_2_ = 124.5	Room temperature	10 °C/min from 0 to 150 °C	[141]
			Exo peak = 105.5			
		Form I	Tm = 123.5			
6P	Dexamethasone	Form A	Tm = 242	Room temperature	5 °C/min	[27]
		Form B	Tm = 250			
7P	Sofosbuvir (anhydrous form 1)	Form 1	Tm = 96.57	Room temperature	0 to 300 °C, 5 °C/min	[79]
		Form A	Tm = 117.90			
		Form B	Tm = 124.83			
		Form V	Tm = 71.54			
8P	Sulindac (form II)	II → I	Endo peak = 160	Room temperature	5 °C/min	[69]
9P	Γ-sorbitol	Γ-sorbitol	Tm = 98.5	Room temperature with dry nitrogen atmosphere	5 °C/min	[75]
		A-form	Tm = 85			
12P	Sulfamerazine	Form I	Tm = 236	Room temperature	100 mL/min	[166]
		Form II	Tm = 212–214			

### 4.4. Identification of Amorphous and Crystalline Phases by Powder X-ray Diffraction (PXRD)

**Figure 3:**
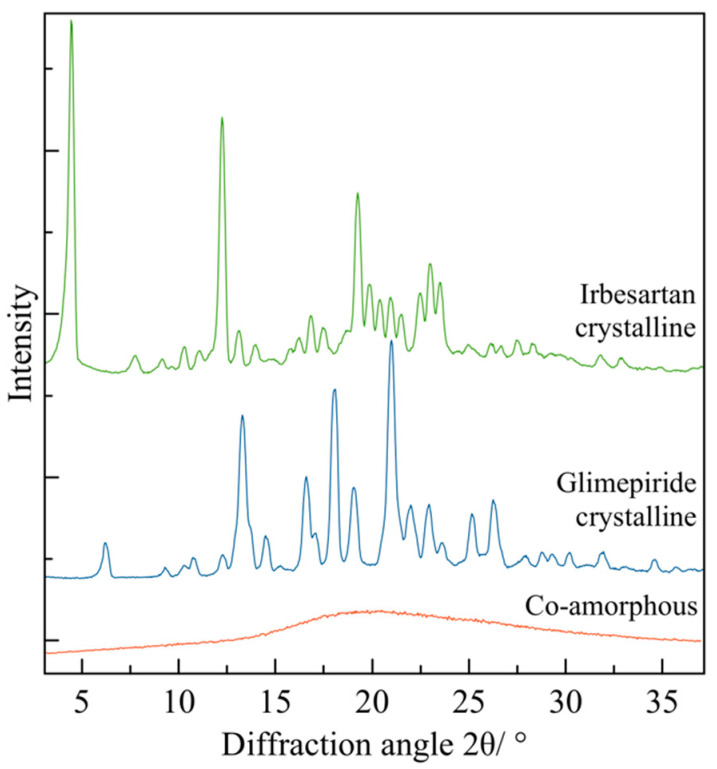
Example of diffractogram of the crystalline pure drug (irbesartan and glimepiride) and co-amorphous form of the binary system.

X-ray diffraction patterns show specific features, depending on the sample analyzed, and allow identification of amorphous and co-amorphous systems, co-crystals, and polymorphs. In this sense, a diffused halo is a clear indicator of the amorphous state (See Figure 3). In addition, XRD allows for identifying specific peaks in co-crystals, differentiation between polymorphs, and degree of crystallinity. In the following, Table 12 and Table 13, the diffraction peaks were directly taken from the articles; when values were not reported, the diffractograms were analyzed in WebPlotDigitizer-3.8 to obtain the accurate values. The samples are marked with an asterisk (*) when data were obtained using this program.

XRD is a technique that can also be useful to identify changes in the crystal system and space groups. Anyway, it allows for the identification of specific peaks that correspond to a particular co-crystal form. From Table 12, it was observed that peaks might vary slightly, depending on the molar ratio [121], and they might even be solvent-dependent [124]. It is worth mentioning that a co-crystal with two polymorphic forms was obtained by Stolar et al. [66] upon the use of mechanochemical preparation (See Row 1 Table 12), but these results will not be further discussed, as they exceed the objectives set out in this review.

Finally, Table 12 also shows that all the articles that reported measurement conditions used a voltage of 40 kV, and the main current used was 40 mA, with step sizes ranging from 0.01 to 0.4, when reported.

A similar analysis can be performed for polymorphs. Each polymorph of a drug shows characteristic diffraction peaks, which enable the accurate identification of the form. It is important to know that milling might cause the disappearance of certain peaks, and new peaks might grow and increase in intensity; this is a clear indicator of the presence of a certain form of the drug (see Table 13).

Besides the information previously discussed, this technique allows analysis of the stability over time of pharmaceutical formulations, which will be discussed below.

(i) Measurement of structural stability on co-amorphous systems during storage by XRD

It is well-known that amorphous samples are not necessarily stable and can recrystallize upon environmental conditions such as high humidity and temperature modification. Table 14 summarizes the information found on articles regarding structural stability, which has been measured under different temperatures ranging from 4 °C to 40 °C, under dry (silica gel and P_2_O_5_) and other humidity conditions (5, 10, and 75% RH) and storage days from 2 to 730 days observing if recrystallization occurred.

More than half of the articles studied structural stability at 25 °C and 40 °C, whereas fewer articles kept the samples at 4 °C or below for further analysis. This stability may depend on the properties of each drug alone, as well as the storage under dry conditions. Note that highly unstable compounds recrystallize immediately after the end of the milling process, even at very low temperatures, such as −15 °C, and a relatively long milling time (14 h) [68]. The reason is that the amorphous state of single drugs is usually less stable (see trehalose dihydrate and α-D-glucose in Table 14) than a co-amorphous system. Therefore, they tend to recrystallize. Nonetheless, other individual drugs studied, such as tadalafil [26] and glibenclamide [89], did not crystallize after 365 and 210 days of storage and 25 °C, respectively. A low percentage of relative humidity rendered amorphous samples for more extended periods.

Badal Tejedor et al. suggest that amorphization is a phenomenon that begins at the surface and propagates to the bulk, thus disrupting the crystalline structure of the material, where additional changes clearly occur at the surface during prolonged milling times [93]. They noticed that other factors can affect the amorphous state’s physical stability once amorphization is reached. These are: (1) remanence of nuclei during milling [167]; (2) different local order in the milled material changes nucleation and growth properties of the crystalline form [95]; and (3) larger specific surface of the milled material can also promote crystallization because the molecular mobility is higher at the surface than in bulk [168].

**Table 14:** Overview of structural stability of amorphous systems upon storage in diverse conditions.

#	Sample	XRD Interpretation	Storage Time (Days)	Storage Conditions *	Ref.
2A	Furosemide-arginine, furosemide-citrulline nitrofurantoin-arginine, nitrofurantoin-citrulline (1:1)	Remained amorphous	450	25 °C, (dry conditions, silica gel)	[85]
	Furosemide-arginine, furosemide-citrulline, nitrofurantoin-arginine	Remained amorphous	450	40 °C, (dry conditions, silica gel)	
	Nitrofurantoin-citrulline	Recrystallization of Nitrofurantoin	450	40 °C, (dry conditions, silica gel)	
3A	Sulfathiazole-polyvinylpyrrolidone sulfadimidine-polyvinylpyrrolidone	Diffused halo → amorphous state	365	4 °C with desiccant	[86]
4A	Naproxen-cimetidine (1:1)	Halo, most stable sample	186	4 °C, 25 °C and 40 °C, dry conditions (silica gel)	[87]
	Naproxen-cimetidine (2:1)	Halo, stable	33	4 °C, dry conditions (silica gel)	
	Naproxen-cimetidine (2:1)	Crystalline naproxen (in excess) peaks	33	25 °C and 40 °C, dry conditions (silica gel)	
	Naproxen-cimetidine (1:2)	Traces of crystalline cimetidine	33	4 °C, 25 °C and 40 °C, dry conditions (silica gel)	
5A	γ-indomethacin–ranitidine hydrochloride (1:1)	Halo, highest stability	30	4 °C and 25 °C, dry conditions (silica gel)	[28]
	γ-indomethacin–ranitidine hydrochloride (2:1)	Small crystalline peaks of indomethacin (indo in excess)	30	25 °C and 40 °C, dry conditions (silica gel)	
	γ-indomethacin–ranitidine hydrochloride (1:2)	Progressive increase in peak intensity as temperature increased.	30	4 °C, 25 °C and 40 °C, dry conditions (silica gel)	
6A	γ-indomethacin	γ-form, crystallized	<1	22 °C over P_2_O_5_	[88]
	α-indomethacin	α-form crystallized to γ-form	4		
7A	Tadafil	Amorphous	365	4 °C with desiccant	[26]
8A	Glibenclamide (GCM)	Broad halo, amorphous state	210	25 °C, 10% RH, dry conditions	[89]
9A	Trehalose dihydrate	Recrystallised material is trehalose dihydrate	2	25 °C	[90]
10A	Atenolol-hydrochlorothiazide (1:1)	Amorphous, stable	30	4 °C and 25 °C, in desiccator	[91]
	Atenolol-hydrochlorothiazide (1:2)	Amorphous, stable	30	4 °C, in desiccator	
	Atenolol-hydrochlorothiazide (1:2)	Traces of crystals	30	25 °C, in desiccator	
12A	Dexamethasone	Form A converts to form B	7	150 °C	[27]
14A	α-D-glucose	Absence of Bragg peaks → amorphization	Immediate analysis after 14 hrs of milling	−15 °C	[68]
		Well-defined Bragg peaks → crystalline state	Immediate analysis after 14 hrs of milling	25 °C	
15A	Mebendazole-ASPA	Amorphous	120 days	25 °C and 40 °C (silica gel)	[94]
	Tadalafil-ASPA	Amorphous	120 days	25 °C and 40 °C (silica gel)	
	Piroxicam-ASPA	Amorphous	120 days	25 °C and 40 °C (silica gel)	
16A	β-D-Glucose	Bragg peaks restore immediately after the end of the milling process	1 h	25 °C	[95]
17A	Carvedilol, carbamazepine, furosemide, indomethacin, mebendazole-amino acids	Recrystallization → Meb-Lys, Meb-Ile, Meb-Leu, Car-Val, Sim-Lys, Ind-Ile, Ind-Val	140	25 °C, 5% RH (P_2_O_5_)	[31]
		Recrystallization peaks → Fur-Met, Fur-Val, Ind-Leu	140–365		
		Amorphous → Arg-Fur, Arg-Ind, His-Fur, Lys-Fur, Lys-Ind, Car-Ile, Car-Leu, Car-Met, Car-Phe, Car-Trp, Meb-Met, Meb-Phe, Meb-Trp, Sim-Phe, Cbz-Trp, Sim-Trp	365–730		
18A	Indomethacin-lysine	Amorphous halo	252 days	DMB, 25 °C (P_2_O_5_) and 40 °C (silica gel), dry conditions	[96]
		Recrystallization → within 25 days it turned into same crystalline form of LAG	10 days	DMB, 25 °C, 75% RH	
		Crystalline form	252 days	LAG, 25° and 40 °C	
23A	Griseofulvin-tryptophan	Amorphous state, no recrystalization detected	365	Silica gel (13–32% RH), vacuum, 23–28 °C	[100]
25A	Mebendazole-tryptophan-phenylalanine	Remained amorphous	90	40 °C, 2% RH (silica gel)	[102]
	Mebendazole-tryptophanphenylalanine	Remained amorphous			
	Mebendazole-phenylalanine-tryptophan	Remained amorphous			
	Mebendazole-aspartate-tyrosine	Remained amorphous			
	Mebendazole-histidine-glycine	Remained amorphous			
	Mebendazole-proline-tryptophan	Remained amorphous			
	Mebendazole-prolinetryptophan	Remained amorphous			
	Mebendazole-tryptophan	Remained amorphous			
	Mebendazole-proline	Recrystallized			
	All samples	Remained amorphous	90	25 °C, 2% RH (silica gel)	
29A	Naproxen-NAP(Na) (2:1)	Recrystallization peaks are visible	7	40 °C, silica gel	[106]
	Naproxen-NAP(Na) (1:1)	Remained amorphous	60		
32A	Simvastatin-lysine	Amorphous	150	4 °C and 0% RH	[108]
		Recrystallization	90	40 °C and 0% RH	
		Recrystallization	56	Ambient temperature and 60% RH	
	Glibenclamide-threonine	Recrystallization	40	40 °C and 0% RH	
	Glibenclamide-serine-threonine	Recrystallization	90		
	Glibenclamide-serine	Amorphous	180		
	Glibenclamide-serine	Amorphous	180	4 °C and 0% RH	
	Glibenclamide-threonine	Recrystallization	44		
	Glibenclamide-serine-threonine	Recrystallization	90		
	Glibenclamide-serine	Recrystallization	150	Ambient temperature and 60% RH	
	Glibenclamide-threonine	Recrystallization	26		
	Glibenclamide-serine-threonine	Recrystallization	90		
33A	Indomethacin, carbamazepine, L-arginine, L-phenylalanine, L-tryptophan and L-tyrosine	Remained amorphous (halo)	180	40 °C, dry conditions (silica gel)	[169]
35A	Carbamazepine-arginine (1:1, 1:2, 1:3, 1:4) carbamazepine-Citric acid-arginine (1:1:1, 1:1:2, 1:1:3)	Amorphous	60	40 °C, silica gel	[110]
36A	Mebendazole (Meb)-glutamate-arginine (crystalline salt), meb-arginine-glutamate (amorphous salt), meb-glutamatearginine, meb-arginineglutamate (dipeptide)	Remained amorphous	180	25 °C, dry conditions (silica gel), 2% RH	[112]
	Meb-glutamate-arginine meb-arginine-glutamate	Recrystallization	180	40 °C, dry conditions (silica gel), 2% RH	
	Meb-glutamatearginine meb-arginineglutamate	Remained amorphous	180		
38A	Glibenclamide-serine glibenclamide-arginine	Samples after storage were similar to the patterns exhibited before the test	180	40 °C and 75% RH	[170]
39A	Rutin-naringin hydrate (all molar ratios), rutin-hesperidin (all molar ratios), rutin-methionine (1:1), rutin-quercetin dihydrate (1:1, 2:1)	Remained amorphous	12 h	Dry and wet conditions	[114]
	Rutin-methionine (1:2 and 2:1)	Small peaks	12 h	Dry conditions	
	Rutin-quercetin dihydrate (1:2)	Small peaks	12 h	Dry and wet conditions	
40A	Gliclazide (Glz)-nifedipine	Crystallized to a physical mixture	3	Ambient temperature, 56% RH	[38]
	Glz-indapamide, Glz-triamterene, Glz-hydrochlorothiazide	Remained amorphous	180		
	Glz-chlorothiazide	Recrystallized	30		
	Glz-indapamide, Glz-triamterene, Glz-hydrochlorothiazide	Remained amorphous	120	Ambient temperature, 98% RH	
	Glz-hydrochlorothiazide	New peaks	30		
	Glz-triamterene	Small peaks	120		
	Glz-benzamidine	New pattern assigned to the salt	30		
42C	Cilexetil-hydrochlorothiazide	Recrystallization	30	4 °C, 0% RH	[116]
	Cilexetil-hydrochlorothiazide-hydroxypropylmethylcellulose acetate succinate type M (HPMCAS)		60		
	Cilexetil-hydrochlorothiazide		15	40 °C, 75% RH	
	Cilexetil-hydrochlorothiazide-HPMCAS (CH50)	Small reflections	90		
	Cilexetil-hydrochlorothiazide-HPMCAS (CH70)		30		
43C	Glibenclamide-quercetin	Remained amorphous	120	4 °C, 0% RH	[111]
		Recrystallization	390		
			10	Room temperature, 60% RH	
			120	40 °C, 0% RH	

* Acronyms: DMB: dry ball milling, LAG: liquid-assisted grinding, RH: relative humidity.

In this sense, several authors prepared the amorphous systems at different molar ratios (see Table 14), and it was clearly observed that the 1:1 preparation allows for the obtention of the structurally most stable ball-milled mixtures from 30 to 186 days, compared to 2:1 and 1:2 molar ratios.

It has been argued that recrystallization prevails at high temperatures, while amorphization prevails at low temperatures due to low molecular mobility [95] in amorphous systems. For preparations that involve molar ratios different than 1:1, the amorphous state stable is maintained at low temperatures (4 °C). However, as the temperature rises in the sample, recrystallization occurs in the form of a progressive increase in peak intensity, where the excess compound is the one that recrystallizes first [28,87,91]. This observation is supported by thermal behavior, as the samples shift the Tg towards the compound present in excess (See Table 9).

Finally, it is important to mention the results obtained by Kasten et al. (2017), as they analyzed two methods of preparation: DMB and LAG. Interestingly, DMB, whether at 25 or 40 °C, under dry conditions, resulted in a stable amorphous form for 252 days of the amorphous salts prepared. On the other hand, increasing relative humidity at 75% and maintaining the temperature at 25 °C caused recrystallization in the sample after 10 days; surprisingly, not into the crystalline form of the initial compounds, instead they transform into LAG peaks of the crystalline salt. This article is relevant for developing novel drugs because it indicates that although recrystallization of the DBM sample might occur, the recrystallization process will not lead to the initial material. Instead, a crystalline salt will be obtained (the same salt as the one prepared by LAG process). This means enhanced solubility over the crystalline drug will be obtained, even after recrystallization. To put this in perspective, 14-fold (crystalline salt), compared to 90-fold, of the co-amorphous salt.

(j) Measurement of structural stability on co-crystals after milling by XRD

Co-crystals have been little studied, compared to amorphous systems. Only a few articles have subjected the samples to stability tests. The reports showed that the storage time ranged from hours to 180 days, where relative humidity conditions higher than 80% caused the partial dissociation of co-crystals [165] (for further details, see Table 15). More articles are needed to reach conclusions regarding the structural stability of co-crystals, but these drug formulations are stable at high relative humidity values (75% RH) and relatively high temperatures (40 °C).

(k) Structural stability on polymorphs after mechanical activation by XRD

The structural stability of polymorphs has been little studied, as well. Only a few articles were found that performed structural stability tests (see Table 16). The range of temperatures was wide, from 25 °C and heating up to 150 °C, where only Kamali et al. [54] reported humidity with a value of 85% RH. The storage time varied from immediate analysis to 150 days, which allowed for studying the transformations from one polymorph to another. In principle, these changes between forms happen due to the metastable states of the drugs because the system looks for the state with the lowest energy and, therefore, changes into a more stable crystalline form.

These results conclude that a wide field in co-crystals and polymorphs, regarding the structural stability of systems, is yet to be studied and understood. It would be worth researching, in more detail, the shelf life of co-crystals and polymorphs with improved solubility and higher stability. These drug formulations could be used in the pharmaceutical industry, due to their superior properties and therapeutic effects.

## 5. Characterization by Microscopy

Finally, other techniques, although rarely mentioned, are also important for the characterization of drug formulations prepared by milling. For instance, scanning electron microscopy is a well-known technique for analyzing the morphologies of the particles. For pharmaceutical compounds, shape, size, and agglomeration are important characteristics for evaluation. According to Badal Tejedor et al. [93], topographical changes at the particle surface after short and longer milling times suggest changes of the particles’ mechanical properties. It would be worth investigating how size and shape affect the stability and behavior of the compound. Amaro et al. used SEM to analyze polymorphs of rivastigmine hydrogen and found different morphologies for forms I (plate-like shape) and II (elongated tetrahedral/needle-like shape). This technique is useful for reinforcing the information obtained from other techniques for the identification of polymorphs [141].

Another common technique for studing the surface mechanical properties, topography, and energy dissipation [171] of a sample is atomic force microscopy (AFM). Badal Tejedor et al. [93] have concluded that crystalline materials show less deformation under an applied pressure with low energy dissipation in AFM, contrary to an amorphous material, which will be more viscous and show higher dissipation, possibly due to the disorder of the atoms in the structure. The presence of both low and high dissipation values across the map would indicate a partially induced surface amorphization [93].

Finally, ultraperformance liquid chromatography (UPLC) is a little used method, but it used to observe the purity of the sample. In this sense, impurities would be present as major or minor intensity peaks in a chromatogram [89], depending on the drug formulation analyzed.

## 6. Concluding Remarks and Future Works

This review focused on characterization results, in order to study different drug formulations, i.e., co-amorphs, co-crystals, and polymorphs, upon milling.

The analyses of experimental milling conditions showed that, in most cases, the milling method is in dry conditions and low or cryogenic temperatures for co-amorphous. Processing times for this kind of formulation ranged from 60 to 180 min. While, for co-crystals, the grinding time reported was shorter, around 30 min, and required solvent-assisted milling at room temperature. For polymorphs, prolonged periods, longer than one hour, were needed to induce structural rearrangement; milling was performed at room temperature in most cases to obtain a polymorph. It is important to note that this information regarding milling times is just an observation of the range of minimum and maximum periods of milling, based on the experimental data reported in the tables. However, parameters such as time, temperature, frequency, and the number of balls are inherent to the material or system, so the effect of milling parameters on the structure change is multifactorial.

Co-amorphous and co-crystal systems that were successfully prepared by milling with enhanced solubility have been widely studied, thus demonstrating the potential of ball milling as a preparation method for drug formulations. Despite the achievements in increases in its solubility, future work is still needed to improve the stability of co-amorphous; additionally, a wide field, regarding the shelf life of polymorphs and co-crystals, is yet to be researched and understood.

Finally, although scaling ball milling to industrial capacities is still a challenge to address, this preparation method represents a non-thermal and advantageous alternative, as it results in drug formulations with enhanced properties.

## Figures and Tables

**Table 4:** Overview of solubility enhancement of amorphous systems prepared by ball milling.

#	Solubility Evaluation (UV, HPLC)	Sample	Ratio/Composition	Solubilty Increment (Folds)	Ref.
2A	HPLC (IDR)	Furosemide-arginine	1:1	38	[85]
		Nitrofurantoin-arginine		20	
3A	UV (IDR)	Sulfathiazole-polyvinylpyrrolidone	Xpvp = 0.7	5.2	[86]
		Sulfadimidine-polyvinylpyrrolidone		26.5	
4A	UV (IDR)	Co-milled naproxen	1:1	4	[87]
		Co-milled cimetidine		2	
7A	HPLC (Solubility)	Tadalafil *	N/A	1.25 (in H2O)	[26]
				0.79 (in 0.1 M HCl)	
				1.35 (Buffer pH = 6.8)	
				1.83 (in water)	
10A	UV (IDR)	Atenolol-hydrochlorothiazide	1:1	12.5	[91]
15A	HPLC (Powder dissolution studies)	Mebendazole-ASPA	1:1	8.13	[94]
		Tadalafil-ASPA		Similar increase to MEB but less pronounced	
		Piroxicam-ASPA		32.1–35	
17A	HPLC (IDR)	Fur-Phe, Fur-Pro, Fur-Trp	1:1	0.9–1.0	[31]
		Fur-Ile, Fur-Leu, Fur-Met, Fur-Val, Ind-Ile, Ind-Leu, Ind-Met, Ind-Phe, Ind-Pro, Ind-Trp, Ind-Val, Meb-Met, Cbz-Trp		1.1–3.0	
		Fur-Arg, Fur-His, Fur-Lys, Ind-Arg, Ind-Lys, Car-Ile, Car-Leu, Car-Met, Car-Phe, Car-Trp, Car-Val, Meb-Ile, Meb-Leu, Meb-Phe, Meb-Trp		3.1–431.8	
18A	HPLC (IDR)	Indomethacin-lysine	1:1	90	[96]
				14	
23A	HPLC (Kinetic solubility studies)	Griseofulvin-tryptophan	1:1	1.19	[100]
25A	HPLC (Dissolution tests)	Mebendazole-histidine-glycine	1:1:1	19	[102]
		Mebendazole-tryptophan-phenylalanine	1:1:1	46	
		Mebendazole-proline-tryptophan	1:1:1	4.3	
29A	UV	Naproxen-NAP(Na)	1:1	2.9	[106]
30A	UV (IDR)	Carvedilol-L-glutamic acid	1:1	12	[107]
		Carvedilol-L-aspartic acid		13	
		Carvedilol-L-glutamic acid		14	
		Carvedilol-L-aspartic acid		2	
31A	Dissolution studies	Indomethacin-arginine	1:1	1.4	[36]
		Indomethacin-phenylalanine		1	
		Indomethacin-tryptophan		1	
33A	HPLC (IDR)	Carbamazepine-arginine-tryptophan *	1:1:1	1.38	[98]
		Carbamazepine-phenylalanine-tryptophan *	1:1:1	1.2	
		Carbamazepine-tryptophan *	1:1	1.08	
		Indomethacin-L-arginine *	1:1	306	
		Indomethacin-L-phenylalanine *	1:1	4.3	
		Indomethacin-L-tryptophan *	1:1	2.4	
		Indomethacin-L-phenylalanine-L-tryptophan *	1:1:1	3.35	
35A	UV	Carbamazepine-citric acid	1:1	2.2	[110]
		Carbamazepine-citric acid-arginine	1:1:1	2.68	
		Carbamazepine-citric acid-arginine	1:1:2	3.28	
		Carbamazepine-citric acid-arginine	1:1:3	3.4	
36A	HPLC	Glibenclamide-serine	1:1	10	[111]
		Glibenclamide-quercetin	1:1	20	
		Glibenclamide-arginine	1:1	19	
		Glibenclamide-arginine-sls	1:1	21	
37A	HPLC	Mebendazole (Meb)-glutamate-arginine (crystalline salt) *	1:1:1	5.2	[112]
		Meb-glutamate-arginine (amorphous salt) *	1:1:1	3.5	
		Meb-arginineglutamate *	1:1	5.16	
		Meb-glutamatearginine *	1:1	4.9	
38A	HPLC	Indomethacin-meglumine *	1:1	18.56	[113]
			1:2	25.39	
			1:4	28	
		Mefenamic acid-meglumine *	1:1	81	
			1:2	108.6	
			1:4	394.3	
		Indomethacin-polyvinylpyrrolidone *	1:1	0.3	
			1:2	0.3	
			1:4	0.48	
		Mefenamic acid-polyvinylpyrrolidone *	1:1	1.6	
			1:2	4	
			1:4	10.6	
41A	UV	Quercetin-arginine *	1:2	21	[115]

Acronym: IDR: intrinsic dissolution rate.

**Table 5:** Overview of solubility enhancement reported for co-crystal drugs.

#	Solubility Evaluation (UV, HPLC)	Sample	Folds	Ref.
3C	In vitro	Ciprofloxacin-thymol (1:2)	4	[118]
5C	UV	Brexpiprazol-catechol (1:1)	2.5	[120]
		Brexpiprazol-succinic acid (1:1)	2.5	
6C	UV	Quercetin-malonic acid (1:2)	1.056	[121]
7C	UV	Paracetamol-trimethylglycine * (1:1)	0.82	[44]
11C	UV	Ciprofloxacin-nicotinic acid (1:1)	20 (in water)	[124]
			1.5	
		Ciprofloxacin-isonicotinic acid (1:1)	20	
			2.5	
13C	HPLC	Acetazolamide-4-aminobenzoic acid * (1:1)	2.5	[67]
			2.17	
15C	IDR	β-lapachone-resorcinol (1:1)	2	[127]
16C	UV	Norfloxacin-nicotinic acid (with EtOH) pH = 3	No change	[128]
		Norfloxacin-nicotinic acid (with EtOH) pH = 6.1	2	
		Norfloxacin-nicotinic acid (with EtOH) pH = 8.5	<2	
17C	UV (Powder dissolution)	Chlorothiazide-DL-proline (w/acetonitrile-water)	1.05	[129]
		Chlorothiazide-L-proline hydrate (w/acetonitrile-water)	Lower value than the initial drug	
		Chlorothiazide-D-proline hydrate (w/acetonitrile-water)		
19C	HPLC (In vitro release test)	Ferulic acid-nicotinamide	2.4	[131]
		Ferulic acid-isonicotinamide	3.1	
		Ferulic acid-urea	1.1	
21C	HPLC	Itraconazole-4-hydroxybenzamide form II (1:2)	225	[133]
		Itraconazole-4-aminobenzoic acid (1:1)	64	

**Table 6:** Overview of structural characterization by spectroscopy of amorphous/co-amorphous drugs obtained by milling.

#	Sample	Analytical Technique	Wavenumber (cm^−1^)/δ (ppm)		Interpretation	Ref.
			Crystalline	Co-Amorphous		
4A	Naproxen-cimetidine	Raman	670 (C-S-C str)	666 cm^−1^	Shift → unknown mechanism of interaction	[87]
			1601 (ring str)	1604 cm^−1^	Shift → solid-state interaction of imidazole ring with naproxen	
5A	γ-Indomethacin–ranitidine hydrochloride	DRIFTS (FT-IR)	1717 and 1692 (C=O)	1723 and 1679	Broadening and shift	[28]
			N/A	1735 cm^−1^	Shoulder appearance	
			N/A	1723 (C=O)	Peak formation → conjugated carbonyl acid system	
			1692 (C=N)	1679 cm^−1^	Shift → larger C=N double bond character or interaction at benzoyl C=O ocurred	
			1620 (aci-nitro C=N str)	1610	Shift → nitro group forming a bond with indomethacin and indirectly reducing the C=N double bond character	
			N/A	1579	Small peak formation → interaction at the amidine moiety	
6A	γ/α-Indomethacin	Raman	N/A	1540 to 1700 and 2930 to 3100 cm^−1^	Large spectral differences → variations in molecular conformation and intermolecular bonding of amorphous forms	[88]
8A	Glibenclamide	FT-IR	3315 (N-H str)	N/A	Abscence of band upon cryomilling	[89]
			1714 (C=O str)	N/A	Loss in intensity but clearly apparent	
			N/A	1637 (C=N str)	New band → conversion of the amide to the imidic acid form	
9A	Trehalose dihydrate	Raman	30–400 (several peaks)	N/A	Presence of only a broad peak (boson) → amorphous material	[90]
			443, 835, 906, and 1449	433, 843, 912, and 1455 cm^−1^	Shift → amorphous transformation	
10A	Atenolol-hydrochlorothiazide	FT-IR	3361 (N-H str) and 3169 (OH str)	3464 and 3357 cm^−1^	Shift	[91]
			1636 (C=O str)	1664 cm^−1^	Shift → formation of intermolecular interactions	
			1317 (-SO2 str)	1327 cm^−1^	Shift → involvement of -SO_2_ in intermolecular hydrogen bonding	
11A	Indomethacin-arginine	FT-IR	1613 (guanidine group)	1603 cm^−1^	Reduction of signal → possibly extremely weak interactions	[92]
			1709 and 1738 cm−1 (C=O)	N/A	Disappearance of peaks → possibly extremely weak interactions	
		ssNMR	159 ppm (guanidine resonance) and 157 ppm (C5)	N/A	Overlap → not easy to identify salt formation	
	Furosemide-arginine	FT-IR	1670 (C=O)	N/A	Decrease of peak → salt formation	
		ssNMR	169 and 173 ppm (C=O)	175 ppm	One broad resonance → similar environments in the mixture. π-π interactions involved	
15A	Piroxicam-ASPA	FT-IR	1377	1392 cm^−1^	Shift → possible interaction between components	[94]
16A	α-D-glucose	Raman	769.2 and 838	N/A	Presence of only the respective vibrational broadened bands → samples free of mutarotation and show anomeric purity	[95]
	β-glucose		896.4	N/A		
18A	Indomethacin-lysine	FT-IR	1713 (C=O str)	N/A	Disappearance of band → suggests ionization and salt formation	[96]
			N/A	1586 and 1561 cm^−1^ (COO-)	Broad peak → ionized carboxyl group for DMB and LAG, respectively	
19A	Mebendazole-tryptophan	FT-IR	1717 (C=O)	1727 cm^−1^	Shift → loss of hydrogen bonds	[97]
	Pioglitazona-tryptophan		2930 (N-H)	1924 cm^−1^	Shift → formation of hydrogen bonds	
20A	Mefenamic acid-NaTC	FT-IR	754 and 776	747 and 769 cm^−1^	Broadening and shift → loss of long-range order	[37]
			888	N/A	Intensity of strong, sharp band decreases	
			1256	1219 cm^−1^	Shift and overlapping with band at 1193 cm^−1^ → changes in the hydrogen bonding network of mefenamic acid on amorphization	
			1329	1319 cm^−1^	Shift → changes in the hydrogen bonding network of mefenamic acid on amorphization	
			1509/1502	1507 cm^−1^	Split peak becomes a broad centered band	
			1648 and 1196	1662 and 1193 cm^−1^	Shift → no evidence for specific API-NaTC interactions; hydrogen bonding interactions can be ruled out	
21A	Indomethacin-arginine	FT-IR	N/A	1590 cm^−1^ (indol)	Peak structure of individual compounds transformed into a broad plateau with a small peak	[98]
			1707 and 1734	N/A	Disappearance of peaks → carboxylic acid vibrations	
			1314 and 1219	1319 and 1222 cm^−1^	Shift (chlorobenzene and indol, respectively) → changes in molecular environment	
22A	(S)-naproxen-L-arginine	FT-IR	N/A	1568 cm^−1^ (C=O)	New broad peak for the LAG sample → carboxyl group ionized	[99]
			N/A	1708 cm^−1^	New band appearance	
			N/A	1543 cm^−1^ (C=O)	New peak with lower intensity compared to LAG sample (DBM formulation)	
			N/A	1679 cm^−1^	Broad shoulder (DMB)	
23A	Griseofulvin-tryptophan	FT-IR	3401 (NH and OH str), 3011 (CH str)	N/A	Enlargement and broadening of bands	[100]
			N/A	3227 cm^−1^	New band appearance	
			1663 (QC, C=O)	1648 cm^−1^	Small displacement → formation of hydrogen bonding interaction	
24A	Naproxen-tryptophan	FT-IR	1369	N/A	Decrease of C=O band due to interactions with NAP	[101]
			1659	1664 cm^−1^	Band transformed into a peak with decreased intensity → interactions involving CO_2_^-^	
	Naproxen-tryptophan-proline		1650–1750	1699 cm^−1^	Transformation into a broad peak	
			1581	1577 cm^−1^ (amide)	Shift of small shoulder	
	Naproxen-arginine		1679 and 1728 cm^−1^	N/A	Disappearance → indicates salt formation	
			1540, 1600–1700	N/A	Reduction of bands (amide and guanidyl) → Supports salt formation	
	Naproxen-arginine-proline		1550 (amide)	1556 cm^−1^	Shift → co-amorphous system	
			1610		Disappearance of band → co-amorphous blend	
26A	Oxaprozin-randomly-methylated-βCD systems	FT-IR	1725	1718 cm^−1^ (OXA carbonyl)	Reduction of intensity and shift → strong solid-state interactions between the components	[103]
27A	Furosemide-arginine	FT-IR	1672 and 1562	N/A	Transformation of bands into shoulders → Salt formation upon co-amorphization	[104]
			1591	1602 cm^−1^	Shift → salt formation upon co-amorphization	
	Indomethacin-arginine		1714 and 1689	N/A	Disappearance of bands → salt formation	
			N/A	1680 and 1500 cm^−1^	Simultaneous formation of a band plateau → Salt formation	
			N/A	1589 cm^−1^	Formation of a small peak → salt formation	
29A	Naproxen-NAP(Na)	FT-IR	1638–1682	1639 cm^−1^	Disappearance of peaks and formation of a broaden single peak	[106]
			1603	1605 cm^−1^	Shift	
			1585–1574	N/A	Peaks weakened and broadened → formation of intermolecular interactions involving carbonyl groups	
		Raman	N/A	747 cm^−1^	Peak broadened and then disappeared → crystallization of NAP and NAP(Na)	
			N/A	742 cm^−1^	Appearance and increase in peak → presence of NAP indicates increasing presence of crystalline NAP	
			N/A	1383 cm^−1^	Small shoulder peak after 10 min → decreased presence of NAP(Na)	
31A	Arginine-indomethacin	FT-IR	N/A	1500–1750 cm^−1^	Formation of a plateau	[36]
				1321 cm^−1^	Presence of peak	
32A	Simvastatin-L-lysine	FT-IR	3442	3350 cm^−1^ (OH)	Broadening → no clear evidence of strong intermolecular interactions between the components	[108]
			1356 and 1319	1350 and 1312 cm^−1^	Shift (aliphatic) → no clear evidence of strong intermolecular interactions between the components	
	Glibenclamide-L-serine		1519	1534 cm^−1^	Shift (NH urea group) → intermolecular interaction	
			1584 (C=O)	1595 cm^−1^	Shift and merging → intermolecular interaction	
34A	L-tryptophan-indomethacin	Raman	N/A	1680 cm^−1^ (C=O)	Appearance and increase in intensity of a broad band → loss of crystalline forms due to changed intermolecular environment	[109]
		FT-IR	1661 and 1582	1609 cm^−1^	Loss of initial bands and formation of broad band	
			495	532 cm^−1^	Peak shift	
35A	Carbamazepine-citric acid-arginine (1:1:1)	FT-IR	1725, 1659, and 1628, 1568 (C=N)	1724, 1659, 1630, and 1573 cm^−1^	Shift of bands. C=O peak weakened and became a shoulder peak → formation of intermolecular interactions between components	[110]
			1659	1678 cm^−1^	Peak strengthened and shifted → intermolecular interactions	
	Carbamazepine-citric acid-arginine (1:1:2)		1659 and 1630	1678 and 1682 cm^−1^	Shift (guanidyl)	
			1568 (C=N)	N/A	Broadening of peak	
	Carbamazepine-citric acid-arginine (1:1:3)		1659 and 1630	1634 and 1636 cm^−1^	Shift (guanidyl) → formation of a stronger interaction with the amide group and/or aromatic ring	
			1568 (C=N)	1559 and 1589 cm^−1^	Formation of a doublet → formation of a stronger interaction with the amide group and/or aromatic ring	
36A	Glibenclamide-quercetin	FT-IR	1713 and 1649 (C=O)	1680 and 1650 cm^−1^	Broadening and shift of peaks → amorphization	[111]
38A	Mefenamic acid-meglumine	FT-IR	N/A	1375 cm^−1^	Formation of a new band → chemical interaction between carbonyl group and secondary amino group of the components	[113]
40A	Gliclazide-triamterene	FT-IR	N/A	3290 (N-H) cm^−1^	Formation of new H bonds	[38]
			1565 and 1530 (NH2)	1570 and 1536 cm^−1^	Shift → formation of new H bonds	
41A	Quercetin-arginine	FT-IR	3400–3200 (OH) cm^−1^	N/A	Loss of intensity → weak intermolecular bonding with the amino acid	[115]
			1645 (C=O)	1654 cm^−1^	Shift → intermolecular H-bonding	
42A	Candesartan cilexetil-hydrochlorothiazide	FT-IR	N/A	1732 cm^−1^	Visualization of band → occurrence of hydrogen bonds between the components	[116]

**Table 7:** Overview of structural characterization by spectroscopy of drug co-crystals obtained by milling.

#	Sample	Analytical Technique	Wavenumber (cm^−1^)		Interpretation	Ref.
			Crystalline	Co-Crystal		
1C	Nicotinamide: L-(+)-ascorbic acid	Raman	104, 146, 666, 1329	93, 133, 631, 1292 cm^−1^	Change form I → form II	[66]
4C	Urea-caffeine	ATR-FTIR	1682 (C=O)	1707	Shift → hydrogen bonding	[119]
			3341 (N-H)	3185	Shift → hydrogen bonding	
			N/A	809	Appearance of a new peak → co-crystal	
5C	Brexpiprazol-catechol (1:1)	Raman	1320.8, 1375.7, 1469.6, 1650.4	1223.4, 1284.1, 1321.47, 1375.2, 1495.4, 1668.3	Shift, decrease in C=O str → hydrogen bonding	[120]
	Brexpiprazol-succinic acid (1:1)		1320.8, 1375.7, 1469.6, 1650.4	1226.8, 1292.2, 1332.6, 1381.6, 1497.4, 1665.7	Shift, decrease in C=O str → hydrogen bonding	
6C	Quercetin-malonic acid	FT-IR	3411 (O-H)	3427 (1:1) and to 3466 cm^−1^ (1:2)	Shift → co-crystal formation	[121]
			1667 and 1612 (C=O)	1638 cm^−1^ (1:2)	Disappearance and shift → co-crystal formation	
7C	Paracetamol-trimethylglycine	FT-IR	1647 (-CONH_2_), 1595, 1506, 1452 (C_6_H_6_), and 804 (-C_6_H_4_-) for PCA. 1400 cm^−1^ (C-N str) and 1323 (-COO-) for TMG.	N/A	No obvious difference in spectra of sample and co-crystal → proton transfer does not occur, no chemical reaction, this confirms co-crystal formation	[44]
		Raman	1643 (C=O), 1605 (C=C), 1364 (C-H), 1229 (-OH, aryl), 1161 (N-H), 850 (C_6_H_6_, aryl), and 789 (C-O)	1629, 1607, 1591, 1371, 1224, 1159, 858, and 774 cm^−1^	Shift and reduction of band intensities → molecular complex is a co-crystal	
			1454 (C-N) and 882 (-COO-)	1443 and 886 cm^−1^	Shift and reduction of band intensities → molecular complex is a co-crystal	
9C	Acetazolamide-4-hydroxybenzoic acid	Raman	N/A	251 (NH, OH), 1694 and 1738 (sci of, CNH and tor -CH3, and C=O, oop bend of ring)	Appearance of peaks → hydrogen bonding interaction leads to co-crystal formation	[123]
			1081 and 1120	N/A	Weak broad peaks → co-crystal	
			910, 1383	947 (N-H, -CH_3_) and 1372 (HC=CH, O-H, C-N) cm^−1^	Shift → co-crystal formation	
			1284		Disappearance → co-crystal formation	
11C	Ciprofloxacin-nicotinic acid/EtOH	FT-IR	N/A	1729 (COOH), 1627 (C=(ketone)), and 3200–2000 (OH)	Presence of bands and OH superimposed by C-H vib, abscence of H bonding → co-crystal formation	[124]
			1589 (asym COO-) and 1375 (sym COO-)	N/A	Stretches of COO → co-crystal formation	
	Ciprofloxacin-isonicotinic acid		1705 (C=O)	1728 cm^−1^	Displacement and increase in intensity	
			1589 (asym COO-)	N/A	Lower intensity and absence of bands attributed to vibrations of H bond → formation of new supramolecular synthons	
12C	Pyrazinamide-diflunisal	Raman	N/A	244 (benzene ring, C-F), 1185 (O-H, HC-CH), 1370 (OH, O=C-O, C-H), 1406 (COH, C-H) and 1750 (C=O, C-O, C-N, C=O, C-C)	Appearance of peaks → hydrogen bonding in COOH-pyridine hetero-synthon leads to co-crystal formation	[125]
			807	N/A	Disappearance → co-crystal formation	
			458 and 1620	449 and 1612 cm^−1^ (C=O, C-O, C-C, O-H, C=OH)	Shift → co-crystal formation	
14C	Acetazolamide, nicotinamide-2-pyridone	Raman	N/A	475, 857 (CH, NH), 928 and 1716 (C=O, N-H, HO-C=O)	Appearance of bands → hydrogen bonding interaction leads to co-crystal formation	[126]
			1014	N/A	Disappearance → co-crystal formation	
			1242, 1456 and 1542	1260 (O=C-N-H, HC=CH), 1466 (-CH3, O=CNH, N-C-H) and 1559 (C-CH, HC=CH, NCH) cm^−1^	Shift → hydrogen bonding interaction leads to co-crystal formation	
16C	Norfloxacin-nicotinic acid	FT-IR	1716 (C=O)	1728 and 1707 cm^−1^	Displacement → New intermolecular interactions	[128]
			N/A	365–2492 cm^−1^	Presence of a broad band → interactions through carboxyl and aromatic nitrogen groups of Nicotinic acid molecules	
17C	Chlorothiazide-L-proline hydrate	FT-IR	N/A	3337 (NH) cm^−1^	Broad peaks → hydrogen bonding	[129]
	Chlorothiazide-D-proline hydrate					
				1332 cm^−1^	Shift → formation of hydrogen bond O-H water -Osulfonamide	
18C	Praziquantel-poloxamer F-127 and sucrose stearate	ATR-FTIR	1625	1621 cm^−1^	Shift → hydrogen bond formation	[130]
20C	Ketoconazole-fumaric acid	FT-IR	1645 (C=O)	1700 cm^−1^	Shift → strong hydrogen bonding	[132]
	Ketoconazole-succinic acid			1714 cm^−1^		
21C	Itraconazole-4-hydroxybenzamide (1:2)	FT-IR	1697 (C=O)	1690 cm^−1^	Shift → participation in hydrogen bonding	[133]
			N/A	3469 (N-H) cm^−1^	More prominent band of form II → higher involvement in hydrogen bonds than form I	
				3111 (C-H) cm^−1^	Sharp peak of form I → asymmetric stretching in both molecules	
	Itraconazole-4-aminobenzoic acid (1:1)			1689 cm^−1^	Shift → participation in hydrogen bonding	
23C	Pyrazinamide-4-aminosalicylic acid	Raman	416, 781, 1055, 1662	366, 893, 1000, 1552, 1637 cm^−1^	New peaks → formation of a co-crystal	[135]
25C	Betulin-terephthalic acid (w/acetone or isopropanol)	ATR-FTIR	NR	3300–3600 (OH) and 1020 (C-O) cm^−1^	Shift → intermolecular hydrogen bonding	[137]

N/A = not applicable, NR = not reported.

**Table 8:** Overview of structural characterization by spectroscopy of drug polymorphs obtained by milling.

#	Sample	Analytical Technique	Wavenumber (cm^−1^)/δ (ppm)		Interpretation	Ref.
			Polymorph I	Polymorph II		
1P	Ranitidine hydrochloride form 1	DRIFTS	1551 (form 1)	1046 (form 2)	Identification of each band → presence of polymorph	[74]
4P	Rivastigmine (RHT form II)	ATR-FTIR	1694 (carbamate, form II)	1725 cm^−1^	Band broadening and shift → form II to I	[141]
6P	Dexamethasone	ssNMR	14–155 ppm (form B)	N/A	Disappearance at high temperatures → change in conformational properties of the molecules and coarsening process.	[27]
10P	Famotidine (form B)	Raman	3406 (N-H str) and 2897 (C-H sym str) (form B)	3455 (N-H str), 3422, 2997 cm^−1^	Clear observation of bands → polymorphic conversion to form A	[142]
			2920 cm^−1^ (form A)	N/A	Increase in peak intensity → presence of form A	
			2897 cm^−1^	N/A	Decrease in peak intensity → form B dropped off	
11P	Gabapentin (GBP) form I, II, III, and IV	FT-IR	3300 (OH str, form I)	N/A	Disappearance → dehydration	[76]
			1660 (C=O, form I)	N/A	Decrease in peak intensity → decrease in hydrogen bonding due to dehydration and polymorphic transformation to II	
			1624 (carboxylate, form I)	1620 cm−1 and then to 1615 cm^−1^	Shift and decrease in peak intensity → decrease in hydrogen bonding due to dehydration and polymorphic transformation to II	
			N/A	1301, 709, 2930, 2153, 1615, 1547, and 1165 (form II)	Appearance of peaks → presence of form II	
			N/A	1699 and 1677 (GBP-lactam)	Appearance of peaks → formation of traces of GBP-lactam due to heating effect	
			N/A	1644, 1584, 1510, 1462, 1400, 1231, 1160, 1512, 2926, and 2200 (form III)	Appearance of specific peaks → presence of form III	
			N/A	3150, 1523, 1397, 1377, 1087, 2121, 1621, 1576, and 1431 (form IV)	Appearance of peaks → presence of form IV	

N/A = not applicable.

**Table 9:** Overview of thermal characterization (DSC) of amorphous samples obtained by ball milling.

#	Sample	Molar Ratio/Composition	Glass Transition Temperature (Tg)/(°C)	Milling Temperature	Conditions	Ref.
2A	Furosemide-arginine	1:1	127 ± 0.5	5 °C	2 °C/min, −10 °C to 180 °C, 50 mL/min	[85]
	Furosemide-citrulline	1:1	77.1 ± 5.6			
	Nitrofurantoin-arginine	1:1	139.1 ± 0.2			
	Nitrofurantoin-citrulline	1:1	49.3 ± 2.1/108.5 ± 0.3			
	Cimetidine-arginine	1:1	40.4 ± 3.1			
	Cimetidine-citrulline	1:1	39.5 ± 1.5			
	Mebendazole-arginine	1:1	53.5 ± 3.3/112.2 ± 0.4			
	Mebendazole-citrulline	1:1	43.6 ± 1.2/112.1 ± 0.2			
3A	Sulfathiazole-polyvinylpyrrolidone	STZ/PVP Xpvp = 0.4	173.2	Room temperature	10 °C/min	[86]
	Sulfadimidine-polyvinylpyrrolidone	SDM/PVP Xpvp = 0.6	146.7			
4A	Naproxen-cimetidine	1:1	34.5	4 ± 2 °C	10 K min^−1^	[87]
		2:1	31.5			
		1:2	40.2			
5A	γ-indomethacin–ranitidine hydrochloride	1:1	32.5	4 ± 2 °C	10 K per min from 0 to 160 °C	[28]
		2:1	34.3			
		1:2	29.3			
6A	γ-indomethacin	N/A	39.23	4 ± 2 °C	10 K min^−1^ from 0 to 180 °C under nitrogen gas flow 50 mL min^−1^	[88]
	α-indomethacin	N/A	37.92			
7A	Tadafil	N/A	147	Cryogenic temperature (liquid nitrogen)	10 °C/min under nitrogen atmosphere (60 mL/min)	[26]
8A	Glibenclamide	N/A	65	Cryogenic temperature (samples immersed in liquid nitrogen)	10 K/min from 20 to 190 °C	[89]
9A	Trehalose dihydrate	N/A	21	Cryogenic temperature (samples immersed in liquid nitrogen)	10 °C/min from 0 to 150 °C	[90]
10A	Atenolol-hydrochlorothiazide	1:1	311.44	Cryogenic temperature (samples immersed in liquid nitrogen)	10 °C/min, starting at −20 °C	[91]
		1:2	315.82			
		2:1	Not determined due to fast recrystallization			
11A	Indomethacin-tryptophan	1:1	Tg ranges from 120 to 45 °C, decreasing as mol% of Ind increases	6 °C	2 K/min from −20 to 180 °C	[92]
	Furosemide-tryptophan	1:1	Tg ranges from 138 to 80 °C, decreasing as mol% of Fur increases			
12A	Dexamethasone	N/A	115 < Tg < 120	Room temperature	0.663 °C and 50 S, “Heat only” conditions	[27]
13A	α-lactose	N/A	70	30 ± 5% relative humidity and 22 ± 3 °C	From 0 to 240°, 10 °C/min under N2 flow of 50 mL/min	[93]
14A	α-D-glucose	N/A	38	−15 °C and 0% relative humidity	5 °C/min, flushed with highly pure nitrogen gas	[68]
15A	Mebendazole-ASPA	1:1	91	5 °C, cold room	−10 °C to 180 °C, 2 °C/min, nitrogen flow was 50 mL/min	[94]
	Tadalafil-ASPA	1:1	102.9			
	Piroxicam-ASPA	1:1	76			
16A	α-D-glucose	N/A	38	−15 °C and 0% relative humidity	5 °C/min	[95]
	β-D-glucose	N/A	39		5 °C/min	
17A	Carvedilol, carbamazepine, furosemide, indomethacin, mebendazole-amino acids	1:1	A single Tg for each formulation	Cold room (+6 °C)	Nitrogen flow of 50 mL/min, 2 °C/min heated to 180 °C	[31]
18A	Indomethacin-lysine	1:1	100 (DMB)	Cold room (+6 °C)	Nitrogen flow of 50 mL/min, 2 °C/min heated to 180 °C	[96]
19A	Mebendazole-tryptophan	Xmeb = 0.1	53.5	Room temperature	−5 °C to 210 °C at 10 °C/min	[97]
	Pioglitazona-tryptophan	Xpgz = 0.1, 150 min	44.9			
22A	(S)-naproxen-L-arginine	1:1	91.9 ± 0.2	6 °C	Nitrogen flow of 50 mL/min, 2 °C/min from −10 °C to 180 °C	[99]
23A	Griseofulvin-tryptophan	1:1	113.46	NR	25 to 300 °C, 5 °C/min	[100]
24A	Naproxen-tryptophan-proline	1:1:1	55.1 ± 3.1	6 °C	Nitrogen flow of 20 mL/min, 10 K/min, from −20 to 170 °C	[101]
	Naproxen-tryptophan	1:1	58.2 ± 0.5			
	Tryptophan-proline	1:1	67.2 ± 6.8			
25A	Mebendazole-tryptophanphenylalanine	1:1:1	107.5 ± 0.2	5 °C	2 °C/min, heating to 180 °C	[102]
	Mebendazole-phenylalaninetryptophan	1:1:1	104.6 ± 0.2			
	Mebendazole-aspartatetyrosine	1:1:1	61.2 ± 0.9			
	Mebendazole-histidineglycine	1:1:1	34.9 ± 1.2/89 ± 0.6			
	Mebendazole-prolinetryptophan	1:1:1	6.5 ± 0.2			
	Mebendazole-tryptophan	1:1	128.7 ± 0.2			
	Mebendazole-proline	1:1	96.9 ± 0.1			
	Mebendazole-proline-tryptophan	1:1:1	56.3 ± 0.2			
	Mebendazole-tryptophan-phenylalanine	1:1:1	119 ± 0.1			
27A	Indomethacin-arginine	1:1	117 ± 4	6 °C	Nitrogen gas flow of 50 mL/min, 2 °C/min, from −10 to 180 °C, 0.212 °C and a period of 40 s	[104]
29A	Naproxen-NAP(Na)	2:1	55.8	4 °C	2 °C/min, 0.2120 °C with a period of 40 s	[106]
		1:1	40			
		1:2	NR			
31A	Indomethacin-arginine	1:1	62.9 ± 0.8	NR	Nitrogen gas flow of 50 mL/min, 10 °C/min to 180 °C	[36]
	Indomethacin-phenylalanine		55.3 ± 0.4			
	Indomethacin-tryptophan		62.7 ± 7.0			
32A	Simvastatin-lysine	1:1	33.2 ± 0.9	6”C	Nitrogen flow of 50 mL/min, 10 °C/min, from −50 °C to 280 °C (depending on the sample)	[108]
	Glibenclamide-serine	1:1	70.1 ± 1.3			
	Glibenclamide-threonine	1:1	58.4 ± 1.3			
	Glibenclamide-serine-threonine	1:1:1	62.5 ± 4.5			
33A	Indomethacin-arginine	1:1	36.7 ± 0.8	6 °C	Nitrogen gas flow, 20 mL/min, from −20 to 180 °C, 10 K/min	[98]
	Indomethacin-phenylalanine	1:1	64.1 ± 1.4			
	Indomethacin-tryptophan	1:1	47.8 ± 2.9			
	Indomethacin-phenylalanine-tryptophan	1:1:1	68.7 ± 2.6			
	Indomethacin-arginine-phenylalanine	1:1:1	63.1 ± 0.8			
	Carbamazepine-tryptophan	1:1	81 ± 0.6		Nitrogen gas flow, 20 mL/min, from −20 to 200 °C, 10 K/min	
	Carbamazepine-phenylalanine-tryptophan	1:1:1	75.1 ± 1.1			
	Carbamazepine-arginine-tryptophan	1:1:1	65.4 ± 1.1			
35A	Carbamazepine-citric acid	1:1	38.8 ± 2.7	4 °C	Nitrogen gas at 50 mL/min, 2 °C/min from 0 to 150 °C, 0.212 °C with a period of 40 s	[110]
	Citric acid-arginine	1:1	56.2 ± 0.7			
	Citric acid-arginine	1:2	106 ± 0.3			
	Citric acid-arginine	1:3	130.5 ± 0.1			
	Citric acid-arginine	1:4	119 ± 0.1			
	Carbamazepine-citric acid-arginine	1:1:1	77.8 ± 1.8			
	Carbamazepine-citric acid-arginine	1:1:2	105.3 ± 0.2			
	Carbamazepine-citric acid-arginine	1:1:3	127.8 ± 0.8			
36A	Glibenclamide-quercetin	1:1	85.97 ± 0.29	Cryomilled	Nitrogen glow of 50 mL/min, 1 °C/min	[111]
37A	Mebendazole-glutamate-arginine (crystalline salt)	1:1:1	37.8	Cold rooms (5 °C)	Nitrogen gas flow of 50 mL/min, 2 °C/min, 0.212 °C (amplitude), 40 s (period)	[112]
	Mebendazole-glutamate-arginine (amorphous salt)	1:1:1	37.3			
	Meb-glutamatearginine	1:1	36.5/77			
	Meb-arginineglutamate	1:1	36.3/76.3			
42A	Candesartan cilexetil-hydrochlorothiazide	NA	110	Room temperature	Nitrogen gas flow, 100 mL/min, 10 °C/min, from 30 to 300 °C	[116]

**Table 10:** Overview of thermal characterization (DSC) of drug co-crystals obtained by ball milling.

#	Sample	Tm Parent Drug 1 (°C) *	Tm Parent Drug 2 (°C)	Tm of Co-Crystal (°C)	Ref.
4C	Urea-caffeine	135.3	235.9	132.7	[119]
5C	Brexpiprazol-catechol	184.8	106.3	161.3	[120]
	Brexpiprazol-succinic acid	184.8	156.1	156.1	
6C	Quercetin-malonic acid	321.92	135.07	283.02 (1:1)	[121]
				266.61 (1:2)	
7C	Paracetamol-trimethylglycine	170.2	320.7	Endo peak = 174.5 °C and 177.4 °C	[44]
11C	Ciprofloxacin-nicotinic acid	254.8	235.1	241	[124]
	Ciprofloxacin-isonicotinic acid	268.3	267.94	242	
13C	Acetazolamide (polymorph I)-4-aminobenzoic acid	269.4	190.5	208.9	[67]
15C	β-lapachone-resorcinol	156	110	131	[127]
16C	Norfloxacin-nicotinic acid (Neat grinding)	222.8	237.1	230.5	[128]
	Norfloxacin-nicotinic acid (LAG)			236.1	
17C	Chlorothiazide-DL-proline	NR	NR	212.9	[129]
18C	Praziquantel-F-127 2B (20:1)	140.23	56.22	133.06	[130]
	Praziquantel-F-127 4B (10:2)			135.97	
19C	Ferulic acid-nicotinamide	172.8	NR	124.6	[131]
	Ferulic acid-isonicotinamide			143.9	
	Ferulic acid-urea			158.1	
20C	Ketoconazole-fumaric acid	151	294	168	[132]
	Ketoconazole-succinic acid		188	164	
21C	Itraconazole-4-aminobenzoic acid *	167	188.5	163.4	[133]
22C	Ibuprofen-nicotinamide	NR	NR	80.5	[134]
24C	Theophylline-4-aminobenzoic acid	274	187	Endos = 161.2 and 168.2	[136]

* Parent drug 1 is the left in the column Sample. Thus, drug parent 2 is on the right.

**Table 12:** Overview of identification of diffraction peaks and measurement conditions for co-crystals.

#	Sample	Co-Crystal	Characteristic Peaks (° 2θ)	Conditions: Current (mA), Voltage (kV), etc.	Ref.
1C	Nicotinamide- L-(+)-ascorbic acid *	Form I polymorph	1.2, 1.5, 1.9, 2.1, 2.8, 3.2, 3.3	7.5 mA, 40 kV	[66]
		Form II polymorph	1.5, 1.8, 2.1, 2.7, 3.1, 3.2		
2C	Salicylic acid-2-pyridone *	sal2hyp	7.8, 11.02, 15.2, 15.8, 16.7, 24.1, 26.8, 28.7	Exposure time 9 s, time separation between patterns 10 s	[117]
	Salicylic acid-3-hydroxypiridine *	sal3hyp	9.2, 20.3, 23.2, 27.5, 31.6		
	Salicylic acid-4-pyridone *	sal4hyp	1.6, 1.9, 2.0, 2.1, 2.8, 3		
3C	Ciprofloxacin-thymol *	N/A	5.3, 7.1, 7.8, 11.4, 13.2, 15.7, 17.51, 19.4, 20.9	40 kV, 40 mA, step size 0.0130°	[118]
4C	Urea-caffeine	N/A	8.64, 10.82, 13.89, 24.30, 25.08, 25.46	35 kV, 25 mA	[119]
5C	Brexpiprazol-catechol	N/A	8.42, 8.88, 11.83, 12.15, 15.75, 16.22	40 kV, 30 mA, step 0.03°	[120]
	Brexpiprazol-succinic acid	N/A	3.67, 9.94, 18.47, 22.25, 22.53, 23.98, 24.3		
6C	Quercetin-malonic acid	CC1 (1:1)	16.21, 19.87, 28.88	40 kV, 40 mA	[121]
		CC2 (1:2)	16.18, 19.86, 28.83		
7C	Paracetamol-trimethylglycine	N/A	17.50, 23.03	40 mA, 40 kV	[44]
8C	Meloxicam-benzoic acid *	N/A	9.2, 12.9, 15.5, 16.7, 20.2, 25.9, 27.3, 28.7, 29.4, 33.1, 35.0	40 kV, 40 mA	[122]
10C	Furosemide-urea *	N/A	7.9, 10.7, 21.1, 26.1, 30.7	Step size 0.017°, collection time 18 h	[51]
11C	Ciprofloxacin-nicotinic acid	CIP-NCA/EtOH (1:1)	9.2, 11.5, 18.5, 19.5, 22.9, 23.4, 26.4, 28.5, 29.4	40 kv, 15 mA, 5–50°, step 0.04°, speed 4°/min	[124]
	Ciprofloxacin-isonicotinic acid	CIP-INCA (without EtOH)	5.4, 10.6, 19.2, 21.4, 28.4		
		CIP-INCA/EtOH	5.4, 10.6		
13C	Acetazolamide-4-aminobenzoic acid *	N/A	6.4, 10.1, 12.1, 12.9, 13.4, 14.1, 15.6, 16.7, 17.2, 17.6, 18.2, 18.3, 19.6, 20.1, 21.4, 22, 23.3, 24.9, 25.6, 26.2, 26.6, 27.8, 29.1	Ambient conditions	[67]
15C	β-Lapachone-resorcinol *	N/A	9.9, 10.5, 11.9, 12.9, 16.8, 18.1, 19.1, 21.4, 21.8, 24.9, 28.8	Speed 1°/min, step size 0.01°	[127]
16C	Norfloxacin-nicotinic acid (with EtOH)	N/A	5.4, 14.5, 25.4	Room temperature, 40 kV, 40 mA	[128]
17C	Chlorothiazide-DL-proline * (w/acetonitrile-water)	N/A	7.3, 20.1, 22.8, 24.12, 25.01	Ambient temperature, 40 kV, 100 mA, 8°/min	[129]
	Chlorothiazide-L-proline hydrate * (w/acetonitrile-water)		8.02, 11.42, 16.4, 23.47, 23.83, 24.95, 25.3		
	Chlorothiazide-D-proline hydrate* (w/acetonitrile-water)		8.2, 11.7, 16.2, 16.7, 17.5, 24.03, 25.2, 26.5, 29.2, 30.9		
18C	Praziquantel-F-127 2B (20:1) *	N/A	8.06, 15.2, 16.4, 16.9, 19.9	40 mA, 40 kV, scan rate 0.02°/s	[130]
	Praziquantel-F-127 4B (10:2) *		6.08, 7.9, 11.9, 12.5, 15.1, 18.8, 19.8, 22.8, 25.3		
20C	Ketoconazole-fumaric acid *	N/A	8.03, 12.2, 16.9, 19.3, 20.3, 21.6, 23.9, 25.7, 28.8	40 kV, 40 mA, step size 0.02°, counting time set 0.2 s/step	[132]
	Ketoconazole-succinic acid *		6.7, 7.9, 12.1, 17.1, 17.7, 19.3, 20.1, 21.2, 23.3, 23.8, 24.3		
21C	Itraconazole-4-hydroxybenzamide form I (1:2) *	N/A	7.3, 9.4, 9.7, 10.3, 11.1, 12.3, 12.7, 16.2, 16.6, 19.3, 20.4, 21.6, 26, 26.3	Ambient conditions, rotated at 15 rpm	[133]
	Itraconazole-4-hydroxybenzamide form II (1:2) *		5.7, 11.4, 12.9, 18.7, 19.04, 21.01, 22.3, 23.8, 25.2		
	Itraconazole-4-aminobenzoic acid (1:1) *		6.1, 10.8, 11.4, 11.9, 13.5, 14, 16.4, 18.8, 19.2, 20.4, 21.2, 21.5, 22, 22.5, 24		
23C	Pyrazinamide-4-aminosalicylic acid	N/A	5.95, 11.91, 13.06, 13.54, 28.25	NR	[135]
24C	Theophylline-4-aminobenzoic acid	N/A	12.3, 14, 15.5, 26.4, 27.5, 28.6	40 kV, 40 mV, step size 0.026° and step time of 56 s	[136]
25C	Betulin-rerephthalic acid (w/acetone) *	N/A	5.08, 8.6, 10.2, 12.8, 14, 14.7, 16, 18.8, 21.3	Range from 5 to 70°	[137]
	Betulin-Terephthalic acid (w/isopropanol) *	N/A	5.1, 8.7, 9.4, 10.2, 12.9, 14.2, 14.6, 16.1, 17.3, 17.9, 18.9, 19.3		

**Table 13:** Overview of identification of diffraction peaks for polymorphs.

#	Sample	Polymorph Identification	Characteristic Peaks (° 2θ)	Ref.
1P	Ranitidine hydrochloride *	Form 1	17, 21.8, 24.9	[74]
		Form 2	20.40, 23.7	
2P	Chlorhexidine dihydrochloride *	Form 1 → initial spectrum	13.9, 18.5, 23.7	[140]
		Form 2 → few peaks	5.2	
		Form 3 → many Bragg peaks	14.9, 28.3	
3P	γ-Sorbitol *	A phase → Sharp peaks, increased milling time	16.6, 30.9	[34]
		γ phase	11.6, 25.5	
4P	Rivastigmine	Form II	9.5, 11.3, 14.2, 15.5, 19.1, 20	[141]
		Form I → Broadeneing of peaks	5.1, 14.7, 16.5, 17.6, 18.6, 20.4, 21.1	
5P	o-Aminobenzoic acid	FI	10.7, 13.7, 14.35, 16.4, 18.6, 23.5, 24.3, 24.9, 26.2, 27.6, 30.5	[54]
		FII	11.2, 15.4, 22.2, 26.7	
	m-Aminobenzoic acid (FIII form)	FI	8.6, 17.2, 24.9	
		FIII	8.3, 16.8, 17.9, 23.7, 23.7, 24.2, 25.9, 26.6, 27.8	
	p-aminobenzoic acid	β-form	17.2, 17.6, 20, 21.9, 25.5, 27.9	
		α-PABA	17.1, 19.9, 21.8, 25.3, 27.8	
6P	Dexamethasone *	Form A	7.9, 13.5, 16.0, 17.6	[27]
		Form B	7.5, 16.8, 18.4	
7P	Sofosbuvir *	Form I	5.3, 7.6, 9.0, 9.8, 10.3	[79]
		Form A	6.2, 8.4, 10.5, 12.8,17.4, 17.9, 18.2, 20.3, 21.1	
		Form B	7.9, 10.3, 12.3, 16.7, 17.1, 19.3, 20, 20.9	
		Form V	5.6, 6.9, 7.5, 10, 10.8, 13.8, 16.4, 19.7, 25.4	
8P	Sulindac *	Form I	10.8, 17.6	[69]
		Form II	9.3, 16.1	
9P	Γ-sorbitol *	Γ-form	11.7, 25.6	[75]
		A-Form	16.7, 31.1	
12P	Sulfamerazine	I	12.6, 14.8, 16.3, 17.4, 20.5, 22.7, 23, 24.6, 31.2, 32.7	[166]
		II	14.5, 17.0, 19.2, 21.5, 26.6, 27.4, 27.9	

**Table 15:** Overview of structural stability of co-crystals upon storage in diverse conditions.

#	Co-Crystal	XRD Interpretation	Storage Time (Days)	Storage Conditions *	Ref.
1C	Nicotinamide-L-(+)-ascorbic acid	Without changes in peaks → chemically stable	180	At shelf	[66]
3C	Ciprofloxacin-thymol	Stable, no changes of crystalline phase	50	Open air	[118]
4C	Urea-caffeine	Formation of co-crystal	Within hours	25 °C, 30% RH	[119]
7C	Paracetamol-trimethylglycine	Physically stable	90	40 and 75% RH	[44]

* Acronym: RH: relative humidity.

**Table 16:** Overview of structural stability of polymorphs upon storage in diverse conditions.

#	Sample	Polymorph Identification	XRD Interpretation	Storage Time (Days)	Storage Conditions	Ref.
5P	o-aminobenzoic acid	Polymorphs: I, II, III, and IV	FII → reappearance of FII	9	25 °C, 40% and 85% RH	[54]
			FII → reappearance of FIII	150	25 °C, 85% RH	
			FI → FII	150	25 °C, 85% RH	
	m-aminobenzoic acid	Polymorphs: I, II, III, IV, and V	FIV	150	25 °C, 85% RH	
			FI → reappearance of FIII	3	25 °C, 85% RH	
	p-aminobenzoic acid	Polymorphs: α and β	β polymorph	150	25 °C, 85% RH	
6P	Dexamethasone	Form A	Broaden Bragg peaks, characteristic of form A	Immediate	Freshly milled samples	[27]
		Form B	Predominantly peaks of form B, peaks of form A decrease	7	Heating up to 150 °C	
7P	Sofosbuvir	Form V	V → transformation to A	120	NR	[79]

Acronym: RH: Relative humidity.

## Data Availability

Data is contained within the article.

## References

[1] Takagi T., Ramachandran C., Bermejo M., Yamashita S., Yu L.X., Amidon G.L. (2006). A Provisional Biopharmaceutical Classification of the Top 200 Oral Drug Products in the United States, Great Britain, Spain, and Japan. Mol. Pharm..

[2] Thayer A.M. (2010). Finding Solutions. Chem. Eng. News.

[3] Kalepu S., Nekkanti V. (2015). Insoluble Drug Delivery Strategies: Review of Recent Advances and Business Prospects. Acta Pharm. Sin. B.

[4] Dengale S.J., Grohganz H., Rades T., Löbmann K. (2016). Recent Advances in Co-Amorphous Drug Formulations. Adv. Drug Deliv. Rev..

[5] Mizoguchi R., Waraya H., Hirakura Y. (2019). Application of Co-Amorphous Technology for Improving the Physicochemical Properties of Amorphous Formulations. Mol. Pharm..

[6] Martínez L.M., Videa M., López Silva T., Castro S., Caballero A., Lara-Díaz V.J., Castorena-Torres F. (2017). Two-Phase Amorphous-Amorphous Solid Drug Dispersion with Enhanced Stability, Solubility and Bioavailability Resulting from Ultrasonic Dispersion of an Immiscible System. Eur. J. Pharm. Biopharm..

[7] Vo C.L.N., Park C., Lee B.J. (2013). Current Trends and Future Perspectives of Solid Dispersions Containing Poorly Water-Soluble Drugs. Eur. J. Pharm. Biopharm..

[8] Zhang X., Xing H., Zhao Y., Ma Z. (2018). Pharmaceutical Dispersion Techniques for Dissolution and Bioavailability Enhancement of Poorly Water-Soluble Drugs. Pharmaceutics.

[9] Tran P.H.L., Tran T.T.D. (2020). Nano-Sized Solid Dispersions for Improving the Bioavailability of Poorly Water-Soluble Drugs. Curr. Pharm. Des..

[10] Dutt B., Choudhary M., Vikaas B. (2020). Cocrystallization: An Innovative Route toward Better Medication. J. Rep. Pharm. Sci..

[11] Berry D.J., Steed J.W. (2017). Pharmaceutical Cocrystals, Salts and Multicomponent Systems; Intermolecular Interactions and Property Based Design. Adv. Drug Deliv. Rev..

[12] Blagden N., de Matas M., Gavan P.T., York P. (2007). Crystal Engineering of Active Pharmaceutical Ingredients to Improve Solubility and Dissolution Rates. Adv. Drug Deliv. Rev..

[13] Llinàs A., Goodman J.M. (2008). Polymorph Control: Past, Present and Future. Drug Discov. Today.

[14] Douroumis D., Ross S.A., Nokhodchi A. (2017). Advanced Methodologies for Cocrystal Synthesis. Adv. Drug Deliv. Rev..

[15] Braga D., Maini L., Grepioni F. (2013). Mechanochemical Preparation of Co-Crystals. Chem. Soc. Rev..

[16] Einfal T., Planinšek O., Hrovat K. (2013). Methods of Amorphization and Investigation of the Amorphous State. Acta Pharm..

[17] Loh Z.H., Samanta A.K., Sia Heng P.W. (2015). Overview of Milling Techniques for Improving the Solubility of Poorly Water-Soluble Drugs. Asian J. Pharm. Sci..

[18] Korhonen O., Pajula K., Laitinen R. (2017). Rational Excipient Selection for Co-Amorphous Formulations. Expert Opin. Drug Deliv..

[19] Han J., Wei Y., Lu Y., Wang R., Zhang J., Gao Y., Qian S. (2020). Co-Amorphous Systems for the Delivery of Poorly Water-Soluble Drugs: Recent Advances and an Update. Expert Opin. Drug Deliv..

[20] Kanaujia P., Poovizhi P., Ng W.K., Tan R.B.H. (2015). Amorphous Formulations for Dissolution and Bioavailability Enhancement of Poorly Soluble APIs. Powder Technol..

[21] Martínez-Jiménez C., Cruz-Angeles J., Videa M., Martínez L.M. (2018). Co-Amorphous Simvastatin-Nifedipine with Enhanced Solubility for Possible Use in Combination Therapy of Hypertension and Hypercholesterolemia. Molecules.

[22] Cruz-Angeles J., Videa M., Martínez L.M. (2019). Highly Soluble Glimepiride and Irbesartan Co-Amorphous Formulation with Potential Application in Combination Therapy. AAPS PharmSciTech.

[23] Martínez L.M., Videa M., López-Silva G.A., de los Reyes C.A., Cruz-Angeles J., González N. (2014). Stabilization of Amorphous Paracetamol Based Systems Using Traditional and Novel Strategies. Int. J. Pharm..

[24] Martínez L.M., Videa M., Sosa N.G., Ramírez J.H., Castro S. (2016). Long-Term Stability of New Co-Amorphous Drug Binary Systems: Study of Glass Transitions as a Function of Composition and Shelf Time. Molecules.

[25] Chavan R.B., Thipparaboina R., Kumar D., Shastri N.R. (2016). Co Amorphous Systems: A Product Development Perspective. Int. J. Pharm..

[26] Wlodarski K., Sawicki W., Paluch K.J., Tajber L., Grembecka M., Hawelek L., Wojnarowska Z., Grzybowska K., Talik E., Paluch M. (2014). The Influence of Amorphization Methods on the Apparent Solubility and Dissolution Rate of Tadalafil. Eur. J. Pharm. Sci..

[27] Oliveira P.F.M., Willart J.-F., Siepmann J., Siepmann F., Descamps M. (2018). Using Milling To Explore Physical States: The Amorphous and Polymorphic Forms of Dexamethasone. Cryst. Growth Des..

[28] Chieng N., Aaltonen J., Saville D., Rades T. (2009). Physical Characterization and Stability of Amorphous Indomethacin and Ranitidine Hydrochloride Binary Systems Prepared by Mechanical Activation. Eur. J. Pharm. Biopharm..

[29] Baláž P., Achimovičová M., Baláž M., Billik P., Cherkezova-Zheleva Z., Criado J.M., Delogu F., Dutková E., Gaffet E., Gotor F.J. (2013). Hallmarks of Mechanochemistry: From Nanoparticles to Technology. Chem. Soc. Rev..

[30] Yu L. (2001). Amorphous Pharmaceutical Solids: Preparation, Characterization and Stabilization. Adv. Drug Deliv. Rev..

[31] Kasten G., Löbmann K., Grohganz H., Rades T. (2019). Co-Former Selection for Co-Amorphous Drug-Amino Acid Formulations. Int. J. Pharm..

[32] Huang Y., Zhang Q., Wang J.R., Lin K.L., Mei X. (2017). Amino Acids as Co-Amorphous Excipients for Tackling the Poor Aqueous Solubility of Valsartan. Pharm. Dev. Technol..

[33] Zhu S., Gao H., Babu S., Garad S. (2018). Co-Amorphous Formation of High-Dose Zwitterionic Compounds with Amino Acids to Improve Solubility and Enable Parenteral Delivery. Mol. Pharm..

[34] Descamps M., Willart J.F., Dudognon E., Caron V. (2006). Transformation of Pharmaceutical Compounds upon Milling and Comilling: The Role of Tg. J. Pharm. Sci..

[35] Wu W., Ueda H., Löbmann K., Rades T., Grohganz H. (2018). Organic Acids as Co-Formers for Co-Amorphous Systems—Influence of Variation in Molar Ratio on the Physicochemical Properties of the Co-Amorphous Systems. Eur. J. Pharm. Biopharm..

[36] Ojarinta R., Heikkinen A.T., Sievänen E., Laitinen R. (2017). Dissolution Behavior of Co-Amorphous Amino Acid-Indomethacin Mixtures: The Ability of Amino Acids to Stabilize the Supersaturated State of Indomethacin. Eur. J. Pharm. Biopharm..

[37] Gniado K., MacFhionnghaile P., McArdle P., Erxleben A. (2018). The Natural Bile Acid Surfactant Sodium Taurocholate (NaTC) as a Coformer in Coamorphous Systems: Enhanced Physical Stability and Dissolution Behavior of Coamorphous Drug-NaTc Systems. Int. J. Pharm..

[38] Aljohani M., MacFhionnghaile P., McArdle P., Erxleben A. (2019). Investigation of the Formation of Drug-Drug Cocrystals and Coamorphous Systems of the Antidiabetic Drug Gliclazide. Int. J. Pharm..

[39] Bansal S., Bansal M., Kumria R. (2012). Nanocrystals: Current Strategies and Trends. Int. J. Res. Pharm. Biomed. Sci..

[40] Babu N.J., Nangia A. (2011). Solubility Advantage of Amorphous Drugs and Pharmaceutical Cocrystals. Cryst. Growth Des..

[41] Kumari N., Ghosh A. (2020). Cocrystallization: Cutting Edge Tool for Physicochemical Modulation of Active Pharmaceutical Ingredients. Curr. Pharm. Des..

[42] Elder D.P., Holm R., De Diego H.L. (2013). Use of Pharmaceutical Salts and Cocrystals to Address the Issue of Poor Solubility. Int. J. Pharm..

[43] Karimi-Jafari M., Padrela L., Walker G.M., Croker D.M. (2018). Creating Cocrystals: A Review of Pharmaceutical Cocrystal Preparation Routes and Applications. Cryst. Growth Des..

[44] Zhao Z., Liu G., Lin Q., Jiang Y. (2018). Co-Crystal of Paracetamol and Trimethylglycine Prepared by a Supercritical CO_2_ Anti-Solvent Process. Chem. Eng. Technol..

[45] Koide T., Takeuchi Y., Otaki T., Yamamoto K., Shimamura R., Ohashi R., Inoue M., Fukami T., Izutsu K. (2020). ichi Quantification of a Cocrystal and Its Dissociated Compounds in Solid Dosage Form Using Transmission Raman Spectroscopy. J. Pharm. Biomed. Anal..

[46] Neurohr C., Revelli A.L., Billot P., Marchivie M., Lecomte S., Laugier S., Massip S., Subra-Paternault P. (2013). Naproxen-Nicotinamide Cocrystals Produced by CO_2_ Antisolvent. J. Supercrit. Fluids.

[47] Müllers K.C., Paisana M., Wahl M.A. (2015). Simultaneous Formation and Micronization of Pharmaceutical Cocrystals by Rapid Expansion of Supercritical Solutions (RESS). Pharm. Res..

[48] Kudo S., Takiyama H. (2014). Production Method of Carbamazepine/Saccharin Cocrystal Particles by Using Two Solution Mixing Based on the Ternary Phase Diagram. J. Cryst. Growth.

[49] Zhou J., Li L., Zhang H., Xu J., Huang D., Gong N., Han W., Yang X., Zhou Z. (2020). Crystal Structures, Dissolution and Pharmacokinetic Study on a Novel Phosphodiesterase-4 Inhibitor Chlorbipram Cocrystals. Int. J. Pharm..

[50] Merah A., Abidi A., Chaffai N., Bataille B., Gherraf N. (2017). Role of Hydroxypropylmethylcellulose (HPMC 4000) in the Protection of the Polymorphs of Piroxicam Extended Release Tablets. Arab. J. Chem..

[51] Al Rahal O., Majumder M., Spillman M.J., van de Streek J., Shankland K. (2020). Co-Crystal Structures of Furosemide: Urea and Carbamazepine: Indomethacin determined from powder X-ray diffraction data. Crystals.

[52] Nugrahani I., Utami D., Ayuningtyas L., Garmana A.N., Oktaviary R. (2019). New Preparation Method Using Microwave, Kinetics, In Vitro Dissolution-Diffusion, and Anti-Inflammatory Study of Diclofenac- Proline Co–Crystal. ChemistrySelect.

[53] Kuang W., Ji S., Wang X., Zhang J., Lan P. (2021). Relationship between Crystal Structures and Physicochemical Properties of Lamotrigine Cocrystal. Powder Technol..

[54] Kamali N., Gniado K., McArdle P., Erxleben A. (2018). Application of Ball Milling for Highly Selective Mechanochemical Polymorph Transformations. Org. Process Res. Dev..

[55] Chieng N., Rades T., Aaltonen J. (2011). An Overview of Recent Studies on the Analysis of Pharmaceutical Polymorphs. J. Pharm. Biomed. Anal..

[56] Cruz-Cabeza A.J., Bernstein J. (2014). Conformational Polymorphism. Chem. Rev..

[57] Cruz-Cabeza A.J., Reutzel-Edens S.M., Bernstein J. (2015). Facts and Fictions about Polymorphism. Chem. Soc. Rev..

[58] Zvoníček V., Skořepová E., Dušek M., Žvátora P., Šoóš M. (2018). Ibrutinib Polymorphs: Crystallographic Study. Cryst. Growth Des..

[59] Stahly G.P. (2007). Diversity in Single- and Multiple-Component Crystals. the Search for and Prevalence of Polymorphs and Cocrystals. Cryst. Growth Des..

[60] Morissette S.L., Soukasene S., Levinson D., Cima M.J., Almarsson Ö. (2003). Elucidation of Crystal Form Diversity of the HIV Protease Inhibitor Ritonavir by High-Throughput Crystallization. Proc. Natl. Acad. Sci. USA.

[61] Lee J., Boerrigter S.X.M., Jung Y.W., Byun Y., Yuk S.H., Byrn S.R., Lee E.H. (2013). Organic Vapor Sorption Method of Isostructural Solvates and Polymorph of Tenofovir Disoproxil Fumarate. Eur. J. Pharm. Sci..

[62] Campeta A.M., Chekal B.P., Abramov Y.A., Meenan P.A., Henson M.J., Shi B., Singer R.A., Horspool K.R. (2010). Development of a Targeted Polymorph Screening Approach for a Complex Polymorphic and Highly Solvating API. J. Pharm. Sci..

[63] Beckmann W., Nickisch K., Budde U. (1998). Development of a Seeding Technique for the Crystallization of the Metastable a Modification of Abecarnil. Org. Process Res. Dev..

[64] Zaccaro J., Matic J., Myerson A.S., Garetz B.A. (2001). Nonphotochemical, Laser-Induced Nucleation of Supersaturated Aqueous Glycine Produces Unexpected γ-Polymorph. Cryst. Growth Des..

[65] Pasquali I., Bettini R., Giordano F. (2008). Supercritical Fluid Technologies: An Innovative Approach for Manipulating the Solid-State of Pharmaceuticals. Adv. Drug Deliv. Rev..

[66] Stolar T., Lukin S., Tireli M., Sović I., Karadeniz B., Kereković I., Matijašić G., Gretić M., Katančić Z., Dejanović I. (2019). Control of Pharmaceutical Cocrystal Polymorphism on Various Scales by Mechanochemistry: Transfer from the Laboratory Batch to the Large-Scale Extrusion Processing. ACS Sustain. Chem. Eng..

[67] Manin A.N., Drozd K.V., Surov A.O., Churakov A.V., Volkova T.V., Perlovich G.L. (2020). Identification of a Previously Unreported Co-Crystal Form of Acetazolamide: A Combination of Multiple Experimental and Virtual Screening Methods. Phys. Chem. Chem. Phys..

[68] Dujardin N., Willart J.F., Dudognon E., Danède F., Descamps M. (2013). Mechanism of Solid State Amorphization of Glucose upon Milling. J. Phys. Chem. B.

[69] Latreche M., Willart J.F., Guerain M., Hédoux A., Danède F. (2019). Using Milling to Explore Physical States: The Amorphous and Polymorphic Forms of Sulindac. J. Pharm. Sci..

[70] Stoler E., Warner J.C. (2015). Non-Covalent Derivatives: Cocrystals and Eutectics. Molecules.

[71] Yamashita H., Hirakura Y., Yuda M., Teramura T., Terada K. (2013). Detection of Cocrystal Formation Based on Binary Phase Diagrams Using Thermal Analysis. Pharm. Res..

[72] Yamashita H., Hirakura Y., Yuda M., Terada K. (2014). Coformer Screening Using Thermal Analysis Based on Binary Phase Diagrams. Pharm. Res..

[73] Ren R., Yang Z., Shaw L.L. (2000). Polymorphic Transformation and Powder Characteristics of TiO_2_ during High Energy Milling. J. Mater. Sci..

[74] Chieng N., Zujovic Z., Bowmaker G., Rades T., Saville D. (2006). Effect of Milling Conditions on the Solid-State Conversion of Ranitidine Hydrochloride Form 1. Int. J. Pharm..

[75] Willart J.F., Lefebvre J., Danède F., Comini S., Looten P., Descamps M. (2005). Polymorphic Transformation of the Γ-Form of D-Sorbitol upon Milling: Structural and Nanostructural Analyses. Solid State Commun..

[76] Lin S.Y., Hsu C.H., Ke W.T. (2010). Solid-State Transformation of Different Gabapentin Polymorphs upon Milling and Co-Milling. Int. J. Pharm..

[77] Friščić T., Trask A.V., Jones W., Motherwell W.D.S. (2006). Screening for Inclusion Compounds and Systematic Construction of Three-Component Solids by Liquid-Assisted Grinding. Angew. Chemie—Int. Ed..

[78] Greco K., Bogner R. (2012). Solution-Mediated Phase Transformation: Significance During Dissolution and Implications for Bioavailability. J. Pharm. Sci..

[79] Chatziadi A., Skořepová E., Rohlíček J., Dušek M., Ridvan L., Šoóš M. (2020). Mechanochemically Induced Polymorphic Transformations of Sofosbuvir. Cryst. Growth Des..

[80] Trask A.V., Shan N., Motherwell W.D.S., Jones W., Feng S., Tan R.B.H., Carpenter K.J. (2005). Selective Polymorph Transformation via Solvent-Drop Grinding. Chem. Commun..

[81] Bouvart N., Palix R.M., Arkhipov S.G., Tumanov I.A., Michalchuk A.A.L., Boldyreva E.V. (2018). Polymorphism of Chlorpropamide on Liquid-Assisted Mechanical Treatment: Choice of Liquid and Type of Mechanical Treatment Matter. CrystEngComm.

[82] Fischer F., Heidrich A., Greiser S., Benemann S., Rademann K., Emmerling F. (2016). Polymorphism of Mechanochemically Synthesized Cocrystals: A Case Study. Cryst. Growth Des..

[83] Gu C.H., Li H., Gandhi R.B., Raghavan K. (2004). Grouping Solvents by Statistical Analysis of Solvent Property Parameters: Implication to Polymorph Screening. Int. J. Pharm..

[84] Kasten G., Grohganz H., Rades T., Löbmann K. (2016). Development of a Screening Method for Co-Amorphous Formulations of Drugs and Amino Acids. Eur. J. Pharm. Sci..

[85] Wu W., Löbmann K., Rades T., Grohganz H. (2018). On the Role of Salt Formation and Structural Similarity of Co-Formers in Co-Amorphous Drug Delivery Systems. Int. J. Pharm..

[86] Caron V., Tajber L., Corrigan O.I., Healy A.M. (2011). A Comparison of Spray Drying and Milling in the Production of Amorphous Dispersions of Sulfathiazole/Polyvinylpyrrolidone and Sulfadimidine/Polyvinylpyrrolidone. Mol. Pharm..

[87] Allesø M., Chieng N., Rehder S., Rantanen J., Rades T., Aaltonen J. (2009). Enhanced Dissolution Rate and Synchronized Release of Drugs in Binary Systems through Formulation: Amorphous Naproxen-Cimetidine Mixtures Prepared by Mechanical Activation. J. Control. Release.

[88] Karmwar P., Graeser K., Gordon K.C., Strachan C.J., Rades T. (2011). Investigation of Properties and Recrystallisation Behaviour of Amorphous Indomethacin Samples Prepared by Different Methods. Int. J. Pharm..

[89] Wojnarowska Z., Grzybowska K., Adrjanowicz K., Kaminski K., Paluch M., Hawelek L., Wrzalik R., Dulski M., Sawicki W., Mazgalski J. (2010). Study of the Amorphous Glibenclamide Drug: Analysis of the Molecular Dynamics of Quenched and Cryomilled Material. Mol. Pharm..

[90] Megarry A.J., Booth J., Burley J. (2011). Amorphous Trehalose Dihydrate by Cryogenic Milling. Carbohydr. Res..

[91] Moinuddin S.M., Ruan S., Huang Y., Gao Q., Shi Q., Cai B., Cai T. (2017). Facile Formation of Co-Amorphous Atenolol and Hydrochlorothiazide Mixtures via Cryogenic-Milling: Enhanced Physical Stability, Dissolution and Pharmacokinetic Profile. Int. J. Pharm..

[92] Jensen K.T., Larsen F.H., Löbmann K., Rades T., Grohganz H. (2016). Influence of Variation in Molar Ratio on Co-Amorphous Drug-Amino Acid Systems. Eur. J. Pharm. Biopharm..

[93] Badal Tejedor M., Pazesh S., Nordgren N., Schuleit M., Rutland M.W., Alderborn G., Millqvist-Fureby A. (2018). Milling Induced Amorphisation and Recrystallization of α-Lactose Monohydrate. Int. J. Pharm..

[94] Wu W., Löbmann K., Schnitzkewitz J., Knuhtsen A., Pedersen D.S., Grohganz H., Rades T. (2018). Aspartame as a Co-Former in Co-Amorphous Systems. Int. J. Pharm..

[95] Dujardin N., Willart J.F., Dudognon E., Hédoux A., Guinet Y., Paccou L., Chazallon B., Descamps M. (2008). Solid State Vitrification of Crystalline α and β-D-Glucose by Mechanical Milling. Solid State Commun..

[96] Kasten G., Nouri K., Grohganz H., Rades T., Löbmann K. (2017). Performance Comparison between Crystalline and Co-Amorphous Salts of Indomethacin-Lysine. Int. J. Pharm..

[97] Martinez L.M., Cruz J. (2018). Preparación de Formulaciones Farmacéuticas Amorfas Usando Metodologías Alternativas Emergentes de Amorfización. https://www.researchgate.net/publication/363611674_PREPARACION_DE_FORMULACIONES_FARMACEUTICAS_AMORFAS_USANDO_METODOLOGIAS_ALTERNATIVAS_EMERGENTES_DE_AMORFIZACION.

[98] Löbmann K., Laitinen R., Strachan C., Rades T., Grohganz H. (2013). Amino Acids as Co-Amorphous Stabilizers for Poorly Water-Soluble Drugs—Part 2: Molecular Interactions. Eur. J. Pharm. Biopharm..

[99] Kasten G., Lobo L., Dengale S., Grohganz H., Rades T., Löbmann K. (2018). In Vitro and in Vivo Comparison between Crystalline and Co-Amorphous Salts of Naproxen-Arginine. Eur. J. Pharm. Biopharm..

[100] França M.T., Marcos T.M., Pereira R.N., Stulzer H.K. (2020). Could the Small Molecules Such as Amino Acids Improve Aqueous Solubility and Stabilize Amorphous Systems Containing Griseofulvin?. Eur. J. Pharm. Sci..

[101] Jensen K.T., Löbmann K., Rades T., Grohganz H. (2014). Improving Co-Amorphous Drug Formulations by the Addition of the Highly Water Soluble Amino Acid, Proline. Pharmaceutics.

[102] Wu W., Löbmann K., Schnitzkewitz J., Knuhtsen A., Pedersen D.S., Rades T., Grohganz H. (2019). Dipeptides as Co-Formers in Co-Amorphous Systems. Eur. J. Pharm. Biopharm..

[103] Mennini N., Maestrelli F., Cirri M., Mura P. (2016). Analysis of Physicochemical Properties of Ternary Systems of Oxaprozin with Randomly Methylated-ß-Cyclodextrin and L-Arginine Aimed to Improve the Drug Solubility. J. Pharm. Biomed. Anal..

[104] Petry I., Löbmann K., Grohganz H., Rades T., Leopold C.S. (2018). In Situ Co-Amorphisation of Arginine with Indomethacin or Furosemide during Immersion in an Acidic Medium—A Proof of Concept Study. Eur. J. Pharm. Biopharm..

[105] Jensen K.T., Larsen F.H., Cornett C., Löbmann K., Grohganz H., Rades T. (2015). Formation Mechanism of Coamorphous Drug-Amino Acid Mixtures. Mol. Pharm..

[106] Ueda H., Peter Bøtker J., Edinger M., Löbmann K., Grohganz H., Müllertz A., Rades T., Østergaard J. (2020). Formulation of Co-Amorphous Systems from Naproxen and Naproxen Sodium and in Situ Monitoring of Physicochemical State Changes during Dissolution Testing by Raman Spectroscopy. Int. J. Pharm..

[107] Mishra J., Löbmann K., Grohganz H., Rades T. (2018). Influence of Preparation Technique on Co-Amorphization of Carvedilol with Acidic Amino Acids. Int. J. Pharm..

[108] Laitinen R., Löbmann K., Grohganz H., Strachan C., Rades T. (2014). Amino Acids as Co-Amorphous Excipients for Simvastatin and Glibenclamide: Physical Properties and Stability. Mol. Pharm..

[109] Walker G., Römann P., Poller B., Löbmann K., Grohganz H., Rooney J.S., Huff G.S., Smith G.P.S., Rades T., Gordon K.C. (2017). Probing Pharmaceutical Mixtures during Milling: The Potency of Low-Frequency Raman Spectroscopy in Identifying Disorder. Mol. Pharm..

[110] Ueda H., Wu W., Löbmann K., Grohganz H., Müllertz A., Rades T. (2018). Application of a Salt Coformer in a Co-Amorphous Drug System Dramatically Enhances the Glass Transition Temperature: A Case Study of the Ternary System Carbamazepine, Citric Acid, and l -Arginine. Mol. Pharm..

[111] Sormunen H., Ruponen M., Laitinen R. (2019). The Effect of Co-Amorphization of Glibenclamide on Its Dissolution Properties and Permeability through an MDCKII-MDR1 Cell Layer. Int. J. Pharm..

[112] Wu W., Grohganz H., Rades T., Löbmann K. (2021). Comparison of Co-Former Performance in Co-Amorphous Formulations: Single Amino Acids, Amino Acid Physical Mixtures, Amino Acid Salts and Dipeptides as Co-Formers. Eur. J. Pharm. Sci..

[113] Slámová M., Prausová K., Epikaridisová J., Brokešová J., Kuentz M., Patera J., Zámostný P. (2021). Effect of Co-Milling on Dissolution Rate of Poorly Soluble Drugs. Int. J. Pharm..

[114] Fujioka S., Kadota K., Yoshida M., Shirakawa Y. (2020). Improvement in the Elution Behavior of Rutin via Binary Amorphous Solid with Flavonoid Using a Mechanochemical Process. Food Bioprod. Process..

[115] Hatwar P., Pathan I.B., Chishti N.A.H., Ambekar W. (2021). Pellets Containing Quercetin Amino Acid Co-Amorphous Mixture for the Treatment of Pain: Formulation, Optimization, In-Vitro and In-Vivo Study. J. Drug Deliv. Sci. Technol..

[116] Pinto J.M.O., Leão A.F., Bazzo G.C., Mendes C., Madureira L.M.P., Caramori G.F., Parreira R.L.T., Stulzer H.K. (2021). Supersaturating Drug Delivery Systems Containing Fixed-Dose Combination of Two Antihypertensive Drugs: Formulation, in Vitro Evaluation and Molecular Metadynamics Simulations. Eur. J. Pharm. Sci..

[117] Lukin S., Stolar T., Tireli M., Barišić D., di Michiel M., Užarević K., Halasz I. (2017). Solid-State Supramolecular Assembly of Salicylic Acid and 2-Pyridone, 3-Hydroxypyridine or 4-Pyridone. Croat. Chem. Acta.

[118] Shemchuk O., Agostino S., Fiore C., Zannoli S., Grepioni F., Braga D. (2020). Natural Antimicrobials Meet a Synthetic Antibiotic: Carvacrol/Thymol and Ciprofloxacin Cocrystals as a Promising Solid-State Route to Activity Enhancement. Cryst. Growth Des..

[119] Macfhionnghaile P., Crowley C.M., McArdle P., Erxleben A. (2020). Spontaneous Solid-State Cocrystallization of Caffeine and Urea. Cryst. Growth Des..

[120] Arabiani M.R., Lodagekar A., Yadav B., Chavan R.B., Shastri N.R., Purohit P.Y., Shelat P., Dave D. (2019). Mechanochemical Synthesis of Brexpiprazole Cocrystals to Improve Its Pharmaceutical Attributes. CrystEngComm.

[121] Setyawan D., Jovita R.O., Iqbal M., Paramanandana A., Yusuf H., Lestari M.L.A.D. (2018). Co-Crystalization of Quercetin and Malonic Acid Using Solvent-Drop Grinding Method. Trop. J. Pharm. Res..

[122] Tantardini C., Arkipov S.G., Cherkashina K.A., Kil’met’ev A.S., Boldyreva E.V. (2018). Synthesis and Crystal Structure of a Meloxicam Co-Crystal with Benzoic Acid. Struct. Chem..

[123] Wang Y., Xue J., Qin J., Liu J., Du Y. (2019). Structure and Spectroscopic Characterization of Pharmaceutical Co-Crystal Formation between Acetazolamide and 4-Hydroxybenzoic Acid. Spectrochim. Acta—Part A Mol. Biomol. Spectrosc..

[124] De Almeida A.C., Torquetti C., Ferreira P.O., Fernandes R.P., dos Santos E.C., Kogawa A.C., Caires F.J. (2020). Cocrystals of Ciprofloxacin with Nicotinic and Isonicotinic Acids: Mechanochemical Synthesis, Characterization, Thermal and Solubility Study. Thermochim. Acta.

[125] Wu X., Wang Y., Xue J., Liu J., Qin J., Hong Z., Du Y. (2020). Solid Phase Drug-Drug Pharmaceutical Co-Crystal Formed between Pyrazinamide and Diflunisal: Structural Characterization Based on Terahertz/Raman Spectroscopy Combining with DFT Calculation. Spectrochim. Acta—Part A Mol. Biomol. Spectrosc..

[126] Fang J., Zhang Z., Bo Y., Xue J., Wang Y., Liu J., Qin J., Hong Z., Du Y. (2021). Vibrational Spectral and Structural Characterization of Multicomponent Ternary Co-Crystal Formation within Acetazolamide, Nicotinamide and 2-Pyridone. Spectrochim. Acta—Part A Mol. Biomol. Spectrosc..

[127] Liu C., Liu Z., Chen Y., Chen Z., Chen H., Pui Y., Qian F. (2018). Oral Bioavailability Enhancement of β-Lapachone, a Poorly Soluble Fast Crystallizer, by Cocrystal, Amorphous Solid Dispersion, and Crystalline Solid Dispersion. Eur. J. Pharm. Biopharm..

[128] Ferreira P.O., de Almeida A.C., dos Santos É.C., Droppa R., Ferreira F.F., Kogawa A.C., Caires F.J. (2020). A Norfloxacin-Nicotinic Acid Cocrystal: Mechanochemical Synthesis, Thermal and Structural Characterization and Solubility Assays. Thermochim. Acta.

[129] Teng R., Wang L., Chen M., Fang W., Gao Z., Chai Y., Zhao P., Bao Y. (2020). Amino Acid Based Pharmaceutical Cocrystals and Hydrate Cocrystals of the Chlorothiazide: Structural Studies and Physicochemical Properties. J. Mol. Struct..

[130] Gaggero A., Jurišić Dukovski B., Radić I., Šagud I., Škorić I., Cinčić D., Jug M. (2020). Co-Grinding with Surfactants as a New Approach to Enhance in Vitro Dissolution of Praziquantel. J. Pharm. Biomed. Anal..

[131] Aitipamula S., Das S. (2020). Cocrystal Formulations: A Case Study of Topical Formulations Consisting of Ferulic Acid Cocrystals. Eur. J. Pharm. Biopharm..

[132] Hossain Mithu M.S., Ross S.A., Hurt A.P., Douroumis D. (2021). Effect of Mechanochemical Grinding Conditions on the Formation of Pharmaceutical Cocrystals and Co-Amorphous Solid Forms of Ketoconazole—Dicarboxylic Acid. J. Drug Deliv. Sci. Technol..

[133] Vasilev N.A., Surov A.O., Voronin A.P., Drozd K.V., Perlovich G.L. (2021). Novel Cocrystals of Itraconazole: Insights from Phase Diagrams, Formation Thermodynamics and Solubility. Int. J. Pharm..

[134] Guerain M., Guinet Y., Correia N.T., Paccou L., Danède F., Hédoux A. (2020). Polymorphism and Stability of Ibuprofen/Nicotinamide Cocrystal: The Effect of the Crystalline Synthesis Method. Int. J. Pharm..

[135] Zhang Z., Fang J., Bo Y., Xue J., Liu J., Hong Z., Du Y. (2021). Terahertz and Raman Spectroscopic Investigation of Anti-Tuberculosis Drug-Drug Cocrystallization Involving 4-Aminosalicylic Acid and Pyrazinamide. J. Mol. Struct..

[136] Shaikh R., Shirazian S., Guerin S., Sheehan E., Thompson D., Walker G.M., Croker D.M. (2021). Understanding Solid-State Processing of Pharmaceutical Cocrystals via Milling: Role of Tablet Excipients. Int. J. Pharm..

[137] Mikhailovskaya A.V., Myz S.A., Bulina N.V., Gerasimov K.B., Kuznetsova S.A., Shakhtshneider T.P. (2019). Screening and Characterization of Cocrystal Formation between Betulin and Terephthalic Acid. Mater. Today Proc..

[138] Da Silva C.C.P., de Melo C.C., Souza M.S., Diniz L.F., Carneiro R.L., Ellena J. (2019). 5-Fluorocytosine/5-Fluorouracil Drug-Drug Cocrystal: A New Development Route Based on Mechanochemical Synthesis. J. Pharm. Innov..

[139] Germann L.S., Arhangelskis M., Etter M., Dinnebier R.E., Friščić T. (2020). Challenging the Ostwald Rule of Stages in Mechanochemical Cocrystallisation. Chem. Sci..

[140] Elisei E., Willart J.F., Danède F., Siepmann J., Siepmann F., Descamps M. (2018). Crystalline Polymorphism Emerging From a Milling-Induced Amorphous Form: The Case of Chlorhexidine Dihydrochloride. J. Pharm. Sci..

[141] Amaro M.I., Simon A., Cabral L.M., De Sousa V.P., Healy A.M. (2018). Rivastigmine Hydrogen Tartrate Polymorphs: Solid-State Characterisation of Transition and Polymorphic Conversion via Milling. Solid State Sci..

[142] Cheng W.T., Lin S.Y., Li M.J. (2007). Raman Microspectroscopic Mapping or Thermal System Used to Investigate Milling-Induced Solid-State Conversion of Famotidine Polymorphs. J. Raman Spectrosc..

[143] Surov A.O., Vasilev N.A., Churakov A.V., Stroh J., Emmerling F., Perlovich G.L. (2019). Solid Forms of Ciprofloxacin Salicylate: Polymorphism, Formation Pathways, and Thermodynamic Stability. Cryst. Growth Des..

[144] Dupont A., Guerain M., Danède F., Paccou L., Guinet Y., Hédoux A., Willart J.-F. (2020). Kinetics and Mechanism of Polymorphic Transformation of Sorbitol under Mechanical Milling. Int. J. Pharm..

[145] Aitipamula S., Chow P.S., Tan R.B.H. (2010). Conformational and Enantiotropic Polymorphism of a 1:1 Cocrystal Involving Ethenzamide and Ethylmalonic Acid. CrystEngComm.

[146] Trask A.V., Motherwell W.D.S., Jones W. (2004). Solvent-Drop Grinding: Green Polymorph Control of Cocrystallisation. Chem. Commun..

[147] Good D.J., Naír R.H. (2009). Solubility Advantage of Pharmaceutical Cocrystals. Cryst. Growth Des..

[148] Alhalaweh A., Roy L., Rodríguez-Hornedo N., Velaga S.P. (2012). PH-Dependent Solubility of Indomethacin-Saccharin and Carbamazepine- Saccharin Cocrystals in Aqueous Media. Mol. Pharm..

[149] Bavishi D.D., Borkhataria C.H. (2016). Spring and Parachute: How Cocrystals Enhance Solubility. Prog. Cryst. Growth Charact. Mater..

[150] Pazesh S., Lazorova L., Berggren J., Alderborn G., Gråsjö J. (2016). Considerations on the Quantitative Analysis of Apparent Amorphicity of Milled Lactose by Raman Spectroscopy. Int. J. Pharm..

[151] Soares F.L.F., Carneiro R.L. (2013). Green Synthesis of Ibuprofen-Nicotinamide Cocrystals and in-Line Evaluation by Raman Spectroscopy. Cryst. Growth Des..

[152] Mukherjee A., Tothadi S., Chakraborty S., Ganguly S., Desiraju G.R. (2013). Synthon Identification in Co-Crystals and Polymorphs with IR Spectroscopy. Primary Amides as a Case Study. CrystEngComm.

[153] Saha S., Rajput L., Joseph S., Mishra M.K., Ganguly S., Desiraju G.R. (2015). IR Spectroscopy as a Probe for C-H⋯X Hydrogen Bonded Supramolecular Synthons. CrystEngComm.

[154] Skorupska E., Kaźmierski S., Potrzebowski M.J. (2017). Solid State NMR Characterization of Ibuprofen:Nicotinamide Cocrystals and New Idea for Controlling Release of Drugs Embedded into Mesoporous Silica Particles. Mol. Pharm..

[155] Apih T., Žagar V., Seliger J. (2020). NMR and NQR Study of Polymorphism in Carbamazepine. Solid State Nucl. Magn. Reson..

[156] Thomas L.C. (2001). Use of Multiple Heating Rate DSC and Modulated Temperature DSC to Detect and Analyze Temperature-Time-Dependent Transitions in Materials. Am. Lab..

[157] Kissi E.O., Kasten G., Löbmann K., Rades T., Grohganz H. (2018). The Role of Glass Transition Temperatures in Coamorphous Drug-Amino Acid Formulations. Mol. Pharm..

[158] Löbmann K., Laitinen R., Grohganz H., Gordon K.C., Strachan C., Rades T. (2011). Coamorphous Drug Systems: Enhanced Physical Stability and Dissolution Rate of Indomethacin and Naproxen. Mol. Pharm..

[159] Gordon M., Taylor J. (1952). Ideal Copolymers and the Second-Order Transition of Rubbers. J. Appl. Chem..

[160] Shamblin S.L., Huang E.Y., Zografi G. (1996). The Effects of Co-Lyophilized Polymeric Additives on the Glass Transition Temperature and Crystallization of Amorphous Sucrose. J. Therm. Anal..

[161] Taylor L.S., Zografi G. (1998). Sugar-Polymer Hydrogen Bond Interactions in Lyophilized Amorphous Mixtures. J. Pharm. Sci..

[162] Masuda T., Yoshihashi Y., Yonemochi E., Fujii K., Uekusa H., Terada K. (2012). Cocrystallization and Amorphization Induced by Drug-Excipient Interaction Improves the Physical Properties of Acyclovir. Int. J. Pharm..

[163] Yamamura S., Gotoh H., Sakamoto Y., Momose Y. (2002). Physicochemical Properties of Amorphous Salt of Cimetidine and Diflunisal System. Int. J. Pharm..

[164] Warner J.C. (2006). Entropic Control in Chemistry and Design. Pure Appl. Chem..

[165] Nugrahani I., Utami D., Ibrahim S., Nugraha Y.P., Uekusa H. (2018). Zwitterionic Cocrystal of Diclofenac and L-Proline: Structure Determination, Solubility, Kinetics of Cocrystallization, and Stability Study. Eur. J. Pharm. Sci..

[166] Zhang G.G.Z., Gu C., Zell M.T., Todd Burkhardt R., Munson E.J., Grant D.J.W. (2002). Crystallization and Transitions of Sulfamerazine Polymorphs. J. Pharm. Sci..

[167] Willart J.F., De Gusseme A., Hemon S., Odou G., Danede F., Descamps M. (2001). Direct Crystal to Glass Transformation of Trehalose Induced by Ball Milling. Solid State Commun..

[168] Desprez S., Descamps M. (2006). Transformations of Glassy Indomethacin Induced by Ball-Milling. J. Non. Cryst. Solids.

[169] Löbmann K., Grohganz H., Laitinen R., Strachan C., Rades T. (2013). Amino Acids as Co-Amorphous Stabilizers for Poorly Water Soluble Drugs—Part 1: Preparation, Stability and Dissolution Enhancement. Eur. J. Pharm. Biopharm..

[170] Sterren V.B., Zoppi A., Abraham-Miranda J., Longhi M.R. (2021). Enhanced Dissolution Profiles of Glibenclamide with Amino Acids Using a Cogrinding Method. Mater. Today Commun..

[171] Tejedor M.B., Nordgren N., Schuleit M., Pazesh S., Alderborn G., Millqvist-Fureby A., Rutland M.W. (2017). Determination of Interfacial Amorphicity in Functional Powders. Langmuir.

